# Modular Europium-Based Nanosensors for the Detection of Sodium and Potassium Ions

**DOI:** 10.1021/acsomega.6c00549

**Published:** 2026-04-20

**Authors:** Adrian A. Mendonsa, Cameron L. Lyman, Logan C. Ruthardt, Raphael Lengacher, M. Andrey Joaqui-Joaqui, Mohammad Al Mesfer, Matt J. Kipper, Eszter Boros, Kevin J. Cash

**Affiliations:** † Department of Chemical and Biological Engineering, 3557 Colorado School of Mines, Golden, Colorado 80401, United States; ‡ Quantitative Bioscience and Engineering Program, Colorado School of Mines, Golden, Colorado 80401, United States; § Department of Chemistry, 5228 University of Wisconsin-Madison, Madison, Wisconsin 53706, United States; ∥ School of Biomedical and Chemical Engineering, 3447 Colorado State University, Fort Collins, Colorado 80523, United States

## Abstract

In this work, we demonstrate a modular approach to ion detection using europium (Eu) chelates as luminescent reporters in Na^+^- and K^+^-selective nanosensors. We evaluated a commercially available option (Eu­(DBM)_3_Phen) and synthetically derived options (Eu­(tacn-PEPA_3_) and Eu­(DBM)_3_Bipy). The sensors exhibited reversible, tunable, and selective responses to their target ions, even in the presence of biologically relevant competing species. Furthermore, Eu­(DBM)_3_Phen showed no degradation when the O_2_ concentration was varied (0–21%) but showed limitations, including a shorter luminescence lifetime (131 μs), pH sensitivity, and susceptibility to interference from divalent cations. In contrast, Eu­(tacn-PEPA_3_) offered a longer lifetime (549 μs), greater pH stability, and reduced ionic interference. To validate platform modularity, the chromoionophore in the sodium sensor was successfully replaced with an alternative dye (BB-dye), confirming that the gating mechanism depends on the chromoionophore selection. These findings underscore the potential of Eu-chelates for biocompatible, time-gated ion sensing, with future work focused on enhancing stability, minimizing pH sensitivity, and shifting excitation wavelengths into the NIR-II region to support in vivo imaging applications.

## Graphical abstract

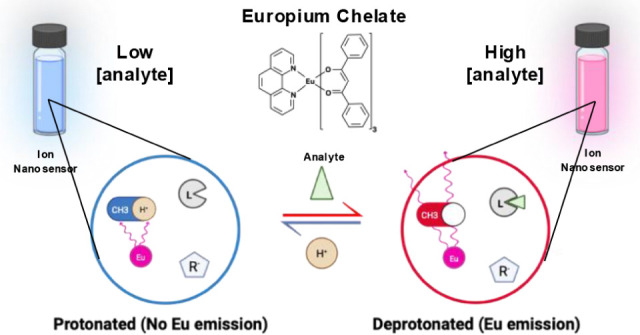

## Introduction

Ions, such as sodium, potassium, and calcium, play crucial roles in assessing the health of both biological and ecological systems.^1,2^ In most aerobic organisms, these ions are involved in regulating pH, transporting water and nutrients into cells, and facilitating nerve signaling.^3–7^ In ecological contexts, potassium serves as an essential micromineral for plant growth and is also used by ecologists as a marker to assess soil and animal health.^8,9^ However, the ability to rapidly and accurately measure analyte concentrations extends beyond biology and ecology, reaching into fields such as food science,^10,11^ chemistry,^12,13^ and agronomy.^8,9^ Ion-selective electrodes (ISEs) are a commonly used tool for ion detection, although they often suffer from issues such as signal drift, fouling, and limited lifespan in complex environments.^14,15^ As a result, optical sensors are gaining traction as a more stable and versatile alternative.

As the name implies, optical sensors rely on optical signals to detect and report changes in analyte levels. Like ISEs, they offer selectivity toward specific analytes but come in a range of formats, including optodes,^16,17^ microneedles,^4^ nanoparticles,^18,19^ thin films,^20,21^ and more. For the purpose of this paper, the focus will be on nanoparticles, primarily due to their high surface area-to-volume ratio, which allows for faster analyte interaction and detection. Their nanoscale size (∼100 nm) also makes them minimally invasive, an important feature for maintaining sterility in biological systems. Additionally, their modular nature allows both the sensing and reporting components to be swapped out, enabling the detection of various analytes, including ions^17,22,23^ and small molecules like oxygen,^18^ ammonium,^24^ and glucose.^25,26^ In a typical polymeric nanosensor, the sensing component is a molecule that selectively binds to the target analyte while the reporter transduces this interaction into a measurable optical signal. In some cases, the sensing and reporter functions are combined in a single compound, as with metalloporphyrins for O_2_ detection. These functional components are typically embedded within a hydrophobic polymeric core, which is encapsulated by a polymeric membrane or surfactant.^27^ The choice of reporter is critical, as it defines the optical output, sensitivity of the sensor, and, in some cases, the selectivity for the analyte of interest. Careful selection or red-shifting of emission wavelengths of the reporter can minimize spectral overlap and interference with competing signals in measured samples. Depending on the system, changes in the analyte can be translated into changes in the emission intensity, wavelength, or lifetime. A range of reporter systems exists, from fluorophores and dyes like carbocyanines and chromoionophores,^28–30^ to advanced materials such as quantum dots,^31,32^ persistent luminescent nanomaterials,^22,33^ metal–organic frameworks (MOFs),^34,35^ and lanthanide-based upconverting particles.^36,37^

Compared to traditional organic fluorophores, lanthanides are an exciting prospect, as they have large energy separation between excitation and emission wavelengths, long luminescent lifetimes, and sharp optical emissions, which make them useful for multiplexing.^38^ Lanthanide ions like europium (Eu^3+^) or terbium (Tb^3+^) emit light through electronic transitions within their 4f orbitals. These transitions are shielded from the environment by outer electron shells, which lead to sharp, well-defined emission peaks. While 4f → 4f transitions are Laporte-forbidden due to parity, they may be partially permitted through the relaxation of selection rules in noncentrosymmetric environments. Sensitization via a ligand allows the complex to overcome the low molar absorptivity of the metal ion, while the symmetry-reducing nature of the surrounding ligands renders these transitions hypersensitive to the local coordination geometry.^39,40^ Furthermore, lanthanide ions (such as Eu^3+^) do not absorb light efficiently by themselves; as a result, chelating ligands are used to protect and enhance the performance of the europium ion. As seen on the left side of Figure 1, upon photon absorption, the ligand is excited from its ground singlet state (S_0_) to a singlet excited state (S_1_), followed by intersystem crossing (ISC) to an excited triplet state (T_1_). The energy of the ligand triplet can either relax radiatively back down to its ground state, be subsequently transferred nonradiatively to the Eu^3+^ excited f-orbital, or, if the energy gap between T_1_ and the metal emitting level is sufficiently small, back energy transfer (BET) may occur, where energy is thermally repopulated from the metal back to the ligand. Ultimately, radiative relaxation from the ^5^D_0_ to ^7^F_2_ level of Eu^3+^ produces the characteristic red emission at ∼612 nm that we associate with Eu-chelates, though nonradiative deactivation pathways (*k*_*nr*_) exist at each stage. However, it is worth noting that this particular emission is impacted by its environment. In short, a chelate or ligand not only improves energy transfer but also shields the ion from quenching interactions with water or other molecules, helping to maintain strong, long-lived emission. Common examples of ligands/chelates used to stabilize lanthanide ions are calixarenes, macrocyclic ligands, β-diketones, carboxylic acid derivatives, and proteins.^41^ For a more in-depth review of lanthanide photophysics and ligands, we recommend the articles by Vuojola and Soukka,^38^ Faulkner et al.,^42^ and Bunzli.^43,44^

**Figure 1:**
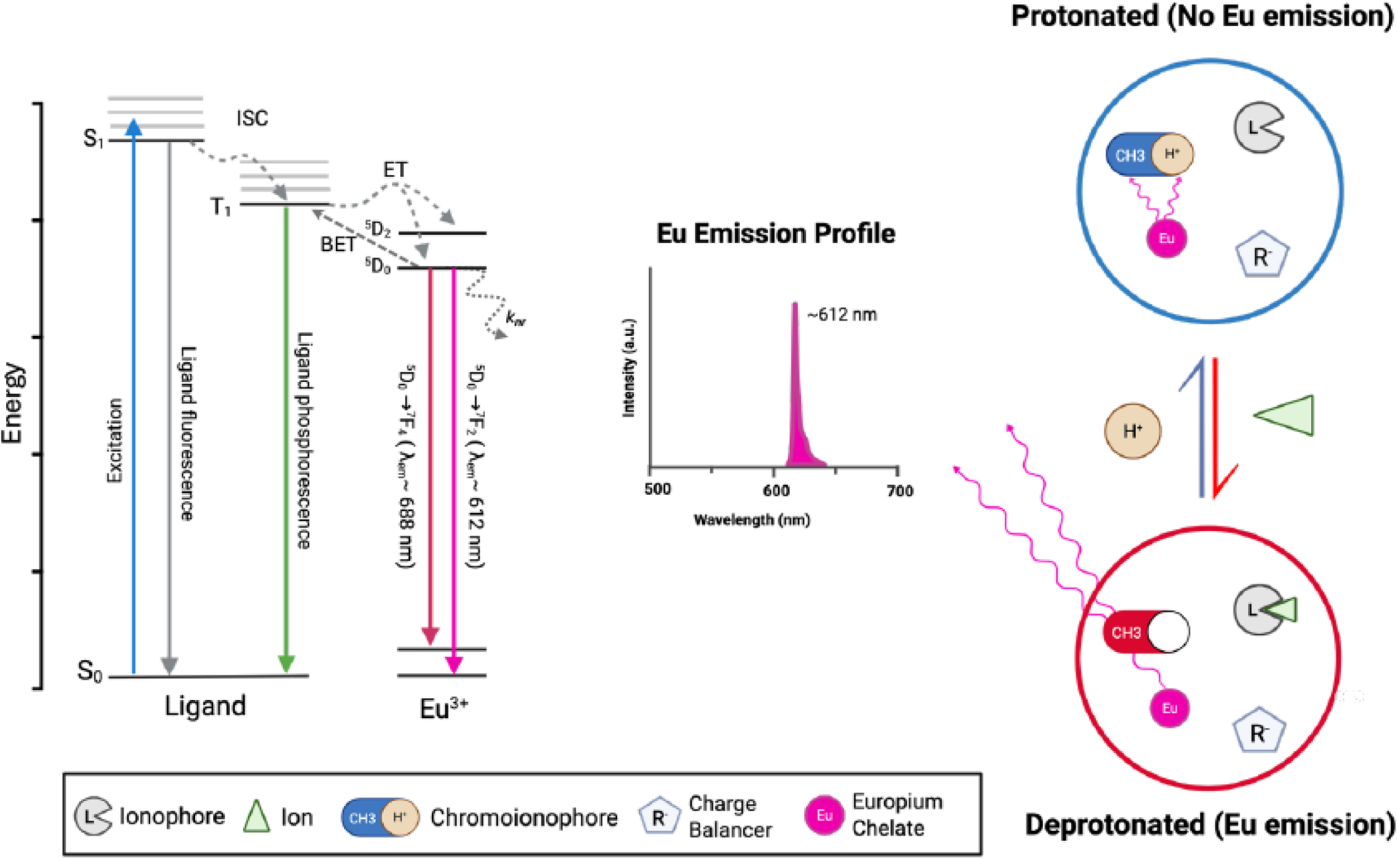
Schematic showing the photophysical pathway for emission from a europium chelate and its operation within an ion-selective nanosensor. When excited with high-energy light, the ligand or chelate helps protect, enhance absorption, and transfer energy to the europium, from which emissions at various wavelengths can occur depending on the ^5^D_0_ → ^7^F_j_ (J = 0–6) transition. The ion-selective sensor operates via an equilibrium-based approach, wherein at low analyte concentrations, the chromoionophore (CH3) is deprotonated (blue color) and has a high absorbance at 612 nm, thus gating the Eu-chelate emission. But when the analyte concentration is high, the chromoionophore protonates to maintain neutrality. As a result, the absorbance at 612 nm decreases, and the Eu-chelate emission increases. Created in BioRender. Mendonsa, A. (2025) https://BioRender.com/5lubfzg.

The use of lanthanides as luminescent reporters or biomarkers is not new to the field of optical sensing. For instance, Liu et al. developed a ratiometric nanosensor to monitor intracellular pH.^45^ They fabricated polymeric hydrogel nanoparticles with a Eu-doped core and a FITC shell (pH-responsive). Their sensor was not only able to quantify the pH of SH-SY5Y cells but also demonstrated good cell viability, signal reversibility, and selectivity against competing cations (Na^+^, K^+^, Ca^2+^, Cu^2+^). Shafabakhsh and coworkers used Eu-chelates on a gold nanoparticle to detect gadolinium-based contrast agents (GBCAs).^46^ Their sensor had an easier workflow and showed higher accuracy than the gold-standard approach (mass spectrometry), potentially giving clinicians a new tool to monitor the excretion of GBCAs post-MRI procedures.^46^ Various groups have demonstrated how Ln-doped MOFs can be leveraged to enable analyte sensing.^34,47,48^ For instance, Yao et al.^47^ developed a Eu-based MOF/PMMA nanocomposite that could reversibly and selectively detect salicylaldehyde vapors in a mixed volatile organic compound environment, while Rozenberga et al.^48^ demonstrated how their structure can simultaneously detect Fe^3+^ and pH. Other groups have used the lanthanide complex itself for applications ranging from imaging, as demonstrated by Lengacher et al.,^49^ to biomarker detection such as H_2_S and amino acids.^50,51^ While it is challenging to comprehensively cover all the techniques, fabrication methods, and applications, the recent review by Alexander et al.^40^ offers a thorough overview of lanthanides and their use in therapeutics and bioapplications ranging from individual molecules to nanoparticles and diagnostic probes.

As highlighted above, the field has developed highly specialized lanthanide sensors, such as NIR-II emitting complexes or MOF-based systems, for analyte detection. Both systems offer unique advantages; for instance, NIR-II Ln complexes are particularly advantageous for analyte detection in complex matrices due to their deep tissue penetration and ability to overcome background autofluorescence.^40^ Similarly, Ln-doped MOFs offer exceptional structural stability and high surface areas for ion interaction.^52^ While effective, these state-of-the-art systems often rely on rigid, highly specialized coordination environments or complex, multistep syntheses that make it difficult to adapt the sensor for multiple analytes without a total structural redesign. In this work, we highlight a different approach, where we exploit shifts in the optical properties of our nanosensors to modulate the signal from the lanthanide chelate, allowing for a modular, plug-and-play approach to ion sensing. Specifically, we exploit shifts in the optical properties of our nanosensors to modulate the signal from the lanthanide chelate. This mechanism is illustrated on the right in Figure 1. In the absence of an analyte, the ionophore remains unbound, the chromoionophore is in its protonated state, and electroneutrality is maintained by the charge balancer. In this state, the emission from the Eu-chelate at λ_*em*_ = 614 nm is minimal, as it is effectively gated. However, when the analyte partitions into the sensing phase, it is extracted by the metal-binding ligand, prompting the chromoionophore to deprotonate.^53,54^ This induces a shift in both luminescence and, more significantly, absorbance. As a result, the gating effect decreases, leading to an increase in the Eu-chelate’s optical emission intensity, as shown in Figure 1. It is important to note that this gating effect is dependent on the specific chromoionophore used. In some cases, such as with Blueberry dye (BB-dye), the opposite effect may occur. To implement this mechanism, we employed a rapid, tunable, and facile fabrication method, flash nanoprecipitation (FNP),^55,56^ to develop a sodium-selective and a potassium-selective nanosensor. These nanosensors utilized two Eu-chelates: a commercially available variant, Eu­(DBM)_3_Phen, and a synthesized analog, Eu­(tacn-PEPA_3_), both of which exhibited sharp, time-resolved emission suitable for ratiometric sensing. We characterized these nanosensors in terms of sensitivity, selectivity, reversibility, stability, and responsiveness to environmental factors, such as pH and dissolved oxygen.

## Experimental Section

### Materials

Tris­(dibenzoylmethane)­mono­(1,10-phenanthroline)­europium­(III) (Eu­(DBM)_3_Phen), tetrahydrofuran (THF, inhibitor-free), potassium ionophore 1 (Selectophore, KI–I), sodium ionophore X (Selectophore, NaI X), sodium tetrakis­[3,5-bis­(trifluoromethyl)­phenyl]­borate (NaBARF), chromoionophore III (Selectophore , CH3), 4-(2-hydroxyethyl)­piperazine-1-ethanesulfonic acid (HEPES), bis­(2-ethylhexyl) sebacate (BEHS or DOS), 1,4,7-triazacyclononane (tacn), europium­(III) chloride hexahydrate (EuCl_3_·6H_2_O, 99.99%), 2,2’-bipyridine (Bipy), dibenzoylmethane (DBM), polystyrene (average MW 35,000 ; PS), and Amicon Ultra centrifugal filter with regenerated cellulose membrane 30 kDa MWCO were purchased from Sigma-Aldrich (St Louis, MO, USA). 6,6’,6’’–((1,4,7-triazonane-1,4,7-triyl)­tris­(methylene))­tris­(4-((4-(2-(2-(2-methoxyethoxy)­ethoxy)­phenyl)­ethynyl)­picolinic acid (pegylated aryl alkyne or PEPA) was synthesized as per Lengacher et al.^49^ Poly­(styrene)-*b*-poly­(ethylene oxide) (PS–PEG or PS_1.6*k*_-*b*-PEO_5*k*_) was obtained from Polymer Source Inc. (Montreal, QC, CA). Blueberry-C6-ester-652 (BB dye) was purchased from Biosearch Technologies (Genomic Analysis by LGC). A 2 M solution (TRIS) and 96-well black-walled optical bottom plates were purchased from Fisher Scientific (Waltham, MA, USA). The confined impinging jet (CIJ) mixer was acquired from Holland Applied Technologies (Burr Ridge, IL). Thirteen kDa MWCO hollow fiber dialysis tubing was purchased from Spectrum Laboratories Inc. (CA, USA).

### Eu-Chelate Synthesis

The Eu­(DBM)_3_Bipy was synthesized according to the method reported by Aleem et al.^57^ Briefly, solutions of EuCl_3_·6H_2_O (0.05 M), DBM (0.15 M), and Bipy (0.05 M) were prepared in absolute ethanol. The DBM solution was heated to 50 °C to fully dissolve the DBM. The solutions of EuCl_3_ and DBM were combined in a 1:3 molar ratio of the solute and stirred for 1 h at room temperature. Then, the pH of the solution was raised to 7–7.5 via dropwise addition of 1 M ammonium hydroxide solution. Finally, an equimolar amount (relative to the Eu^3+^ ion) of the Bipy solution was added, and the reaction mixture was stirred for an additional 3 h at room temperature. The resulting product solution was clear and colorless. The synthesis protocol for the Eu­(tacn-PEPA_3_) is outlined by Walton et al.^58^ Characterization of both these complexes is found in Figures S1 and S2 of the Supporting Information.

### Optode Formulations

The optodes for the potassium and sodium sensors had similar preparation methods. The exact components and their values are detailed in Table S1 of the Supporting Information. Briefly, the appropriate amount of NaBARF and the respective ionophores (NaI X or KI–I) were dissolved in an arbitrary amount of THF and transferred to a 5 mL glass vial, where they were allowed to dry. Subsequently, PS–PEG, PS, CH3, and the Eu-dye were added to the same vial. Note that the PS–PEG (40 mg/mL), PS (13 mg/mL), CH3 (0.005 mg/mL), and Eu-dye (4 mg/mL) were all suspended in THF, and a small aliquot was extracted from the stock solution. Additional THF was then added to bring the total THF volume to 0.5 mL. Finally, BEHS was introduced to the mixture, and the vial was vortexed thoroughly for 10–30 s to ensure complete mixing before being used to fabricate the nanosensors.

### Nanosensor Fabrication

The nanosensors were prepared using an adapted version of the FNP method, previously described by Mendonsa et al.^59^ Instead of PBS, 525 μL of HEPES/Tris (H/T) buffer (pH 7.4) was used as the antisolvent, and 4 mL of the same buffer was added to the quench bath. After fabrication, the nanosensors were left to mix for 10 min on a stir plate, then exposed to a gentle stream of air for 35 min to evaporate any residual THF. The nanosensors were then transferred into a 5 mL glass vial, and additional H/T buffer was added to bring the final volume to 5 mL, compensating for any buffer loss during drying. Finally, the nanosensors were stored in a cool, dark place until further use.

### Nanosensor Characterization

The performance of the nanosensors was characterized by using a Synergy H1 microplate reader (BioTek, now part of Agilent Technologies). Selectivity and sensitivity were evaluated against competing analytes (Na^+^, K^+^, and Ca^2+^) across a broad range of concentrations. The nanosensors were mixed with analyte solutions in a 1:1 volume ratio (v/v) in a 96-well, clear-bottom, black-walled optical plate, yielding final analyte concentrations spanning from 1 M to 1 μM. The acid and base end points were conducted in 0.2 N HCl and 0.2 N NaOH under the same conditions, unless stated otherwise. Both the absorbance and luminescence responses were measured. Luminescence was recorded at three excitation/emission wavelength pairs, with and without a 200 μs time delay. Two channels were used to monitor the chromoionophore (Ex: 500 nm; Em: 585 nm and Ex: 650 nm; Em: 680 nm) and one channel for the Eu-dye (Ex: 352 nm; Em: 614 nm). Absorbance spectra were collected simultaneously from 350–700 nm. A ratio of the signals was taken to generate a calibration curve, which was used to then quantify the nanosensors’ sensitivity and selectivity. For the chromoionophore-only (CH3) ratiometric response, the emission at 680 nm was divided by the signal at 585 nm. Whereas, for the mixed response, CH3’s 680 nm emission was divided by the Eu-dye emission at 614 nm. Ratiometric data were plotted against the analyte concentration and fit to a four-parameter logistic model on Graphpad Prism. Note, due to the pH sensitivity of the Eu-dye, some of the data were normalized to the lowest analyte concentration (1 μM) for comparative purposes.

To evaluate oxygen sensitivity, Eu-dye-containing nanosensors were subjected to varying oxygen levels following protocols described in prior work.^60,61^ Briefly, air and nitrogen were mixed at controlled flow rates and bubbled through a needle into a quartz cuvette to adjust the dissolved oxygen concentration from 0% to 21%. The sample was excited by using a 365 nm LED (ThorLabs), and emissions were recorded by using an Avantes StarLine spectrometer equipped with a 50 μm slit width. The same system, excluding the O_2_ bubbler, was used to obtain the potassium nanosensor’s atomic emission spectra. For these measurements, 500 μL of nanosensor solution was mixed in a 1:1 (v/v) ratio with K^+^ dilutions ranging from 2 M to 2 μM.

Reversibility of the nanosensor response was assessed following the protocol by Galyean et al.^62^ and Sodia et al.^22^ Potassium nanosensors were concentrated 5× using 30 kDa MWCO filters and loaded into 13 kDa MWCO hollow fiber microdialysis tubing. The ends of the tube were sealed onto a clear glass slide with vacuum grease (Dow Corning, Amazon.com), and 50 μL of H/T buffer was added to the tubing prior to imaging on a Fluoview FV10i Confocal Microscope (Olympus, Waltham, MA). The nanosensors were excited at 400 and 635 nm (laser intensity: 80%), and emissions were collected in single-shot mode at 604 nm (Qdot605) and 661 nm (TOTO-3). To change the nanosensor environment, the analyte solution was wicked away using a Kimwipe and replaced with either H/T buffer or 1 M KCl, depending on the condition used in the previous run. Each solution was incubated for 10 min before imaging. Finally, the nanosensors’ functional and colloidal stability were evaluated following established protocols.^59,61^ For these measurements, the nanosensors were diluted to 20% (v/v) in water prior to dynamic light scattering (DLS) and ζ-potential analysis. All samples were taken in triplicate unless stated otherwise.

## Results and Discussion

The field of ion sensing is expansive, with sensors available in various formats and emission wavelengths. However, many of the fluorophores commonly used in these systems suffer from broad emission spectra, which can complicate signal deconvolution and reduce the signal-to-noise ratio (SNR). Additionally, their short luminescence lifetimes preclude them from being used in advanced imaging techniques, such as time-gated imaging/detection.

To address current limitations in ion sensing, we developed two new Na^+^- and K^+^-selective ion sensors using europium (Eu)-based reporters, which are known for their sharp optical emissions and long luminescent lifetimes.^38,40^ These sensors were fabricated using FNP, a rapid and scalable method our group has adapted for nanosensor production.^22,55,61^ In selecting Eu-chelates for these sensors, we focused on a few options: a commercially available complex, Eu­(DBM)_3_Phen, and synthesized variants, Eu­(tacn-PEPA_3_) and Eu­(DBM)_3_Bipy. The choice of using Eu­(DBM)_3_Phen was motivated by the fact that many existing commercial Eu-chelates, such as Eu–N1-DTA chelates,^63^ europium cryptate labels,^64^ europium-labeled streptavidin^65^ (Revvity), Eu-doped FluoSpheres^66^ (Thermo Fisher), and carboxylated Eu-chelate nanoparticles^67^ (Bangs Laboratories), can be prohibitively expensive or have large particle sizes (typically around 100 nm), which can make their incorporation into nanosensors difficult. In contrast, the Eu­(DBM)_3_Phen we use offers a cost-effective and modular alternative for use in a nanosensor platform.

We evaluated the sensitivity and selectivity of sodium and potassium sensors containing the commercial europium complex Eu­(DBM)_3_Phen against a broad analyte concentration range (1 μM to 1 M), which encompasses the biologically relevant concentrations for most aerobic organisms.^68^ Sodium and potassium were the primary/target analytes for the sodium and potassium sensors, respectively, with the other ions acting as competing analytes. As seen in Figure 2, the sensors demonstrated ion-selective responses. The sodium sensor exhibited a detection range from 1 mM to 1 M, with a midpoint response (logEC50) of −1.80 and a favorable selectivity coefficient (log*K* ^Osel^Na,K = −1.61). The potassium sensor, in contrast, had a larger dynamic range (10 μM to 0.1 M) and a lower midpoint response (logEC50) = −3.26), with excellent selectivity (log*K* ^Osel^K,Na = −3.31). For all of the midpoint response and selectivity coefficient values, see Table S2 in the Supporting Information. The sodium sensors containing Eu­(DBM)_3_Phen were also tested against other biologically and environmentally relevant cations and anions such as Fe^3+^, Zn^2+^ and Br^–^, SO_4_^2–^, and Cl^–^ (see Figures S3 and S4). As expected, Fe^3+^ quenched the emission of the europium dye.^69^ This is likely due to Fe^3+^ disrupting the solvation shell around the europium chelate or potentially displacing the metal from the ligand scaffold.^69^ A similar trend was observed when tested against Zn^2+^, where the ratiometric signal increased with increasing concentration (Figure S3). While unexpected, it is explained by the fact that ZnCl_2_ forms a mildly acidic solution^70^ at higher concentrations (>1 mM), which can influence the protonation degree of the CH3 and therefore the sensors’ response. Conversely, varying the background anions had a negligible impact on performance. This stability was expected, as the ionophore and charge balancer work in tandem to maintain cation affinity while effectively excluding anions from the sensing phase (see Figure S4).

**Figure 2:**
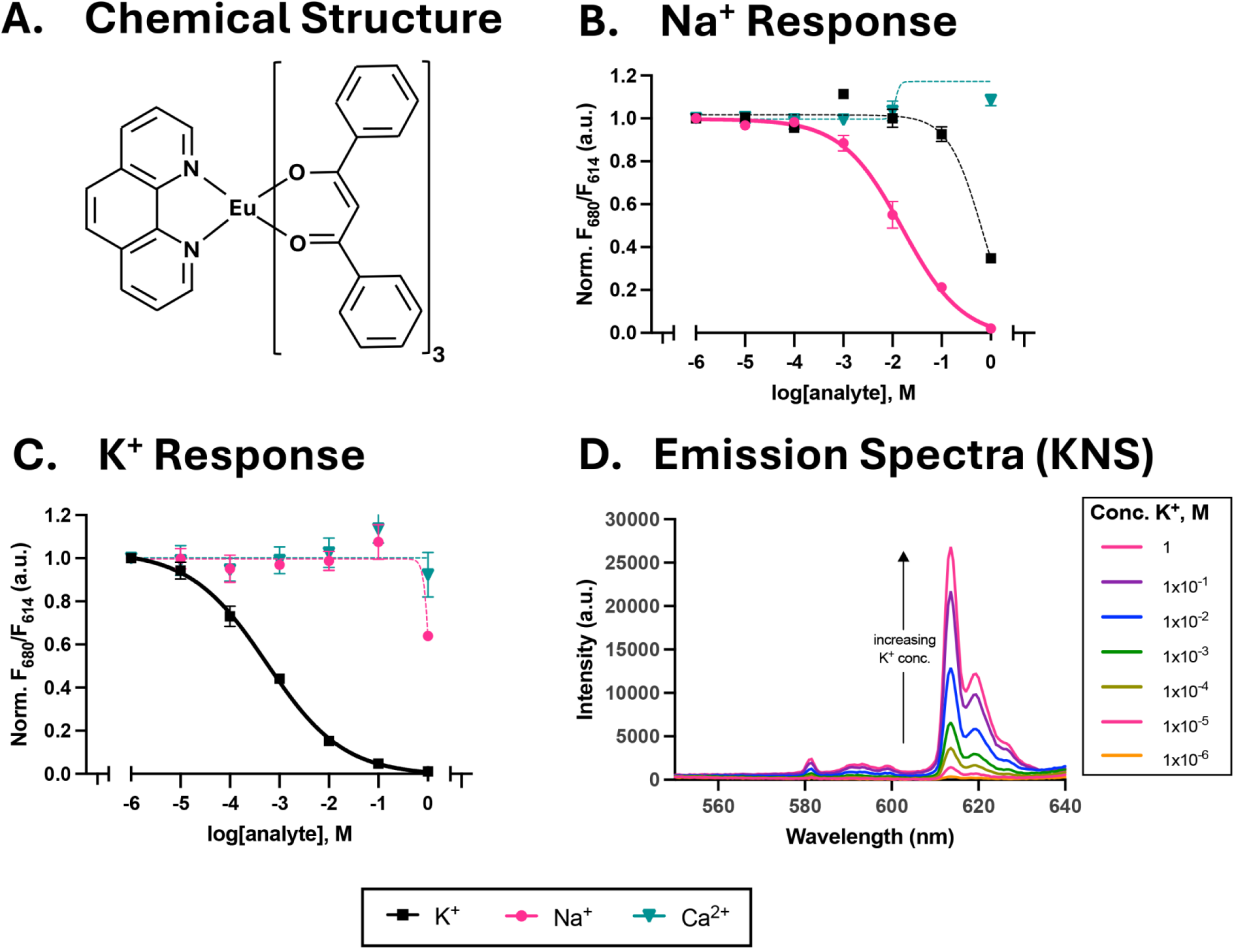
Ion-selective nanosensor performance using an Eu­(DBM)_3_Phen reporter. (A) Chemical structure of Eu­(DBM)_3_Phen, where the dibenzoylmethane (DBM) and 1,10-phenanthroline (Phen) stabilize the europium ion. (B) Normalized ratiometric response of the sodium sensors showing selectivity to sodium ions over competing analytes. (C) Normalized ratiometric response of the potassium sensor showing affinity to potassium over other competing analytes. (D) Emission spectra of the Eu-chelate (Ex: 365 nm) in the potassium nanosensor, upon the addition of increasing amounts of K^+^ with increasing analyte concentration. Note, both ratiometric signals in (B) and (C) were taken by dividing the CH3 signal at 680 nm by the Eu-chelate emission at 614 nm and then normalizing them to the response at 1 μM analyte. Where not visible, error bars are smaller than the data points. The curves in parts B and C are fits to a four-parameter logistic equation.

The underlying mechanism for these sensors is a chromoionophore-mediated gating effect. As the analyte binds to the ionophore, the chromoionophore (CH3) deprotonates, altering its optical properties (luminescence and absorbance). This change in absorbance, in turn, modulates the Eu-chelate’s observed emission, effectively gating its luminescence. Profiling the Eu­(DBM)_3_Phen emission as a function of analyte concentration confirms this, as CH3 absorbance in the 600–700 nm region decreases (see Figure S5), while the Eu-emission at 614 nm increases, as seen in Figure 2D.

Apart from their narrow optical emission, Eu-chelates also have long emissive lifetimes.^38,43^ Depending on the structure, these lifetimes can vary greatly, from 100 μs to nearly 1 ms,^42,44,71^ which often allows them to be used for time-gating applications. Using an Eu-chelate-only sensor (no CH3 or sensing components), we characterized the emission lifetime of Eu­(DBM)_3_Phen which was 131 μs, longer than that of CH3, a commercially available organic fluorophore (Figure S6), or quantum dots, whose emission lifetimes are tens of nanoseconds.^69^ Additionally, when profiled at a time delay of 150 μs, the CH3 signal was no longer detectable, confirming the utility of these constructs for time-gated imaging (Figure S7).

In addition to being selective and sensitive, the sensors also need to be reversible and stable. As shown in Figure 3, we used microdialysis fibers to profile the reversibility of our Eu­(DBM)_3_Phen-based potassium sensors. We used this approach because it allows the analytes to interact with the nanosensors in the fiber without the risk of nanosensors diffusing into the bulk solution^22,62^ and avoids the need for a tedious titrative procedure.^72,73^ The analyte solution was alternated between 1 M KCl and HEPES/Tris (H/T) buffer for two consecutive cycles. As expected, Eu­(DBM)_3_Phen (Figure 3A) showed a decrease in luminescence when the analyte was switched from 1 M KCl to H/T buffer. Conversely, the CH3 signal increased in H/T buffer compared to 1 M KCl (Figure 3B), with the ratiometric signal CH3/Eu demonstrating a reversible response. While effective for most ion applications, the one caveat we faced using this method is the change in temperature inside the confocal microscope during imaging (from 26 to 37 °C). This change in temperature is most evident in the large error bars for the Eu-channel compared to the CH3 channel (Figure S8). This could be explained by the fact that most lanthanides, including this one, are temperature sensitive.^74,75^ When the ambient temperature was increased from a baseline of 25 °C to 35 °C and 45 °C, the Eu­(DBM)_3_Phen signal decreased by 46% and 70%, respectively (compared to the baseline). However, when the temperature was lowered back down to 25 °C, 97% of the baseline signal was recovered (see Figure S9). As a result, the Eu-channel sees a larger variation than the CH3 channel, which is not as temperature sensitive. To mitigate this issue, we limited imaging and equilibration times to under 20 min between each analyte across all cycles. While temperature-dependent sensor output remains a widespread challenge in the field, various strategies, such as calibration curves^76–78^ or specialized temperature-sensitive approaches,^79^ could be employed to account for thermal interference in sensor readings.

**Figure 3:**
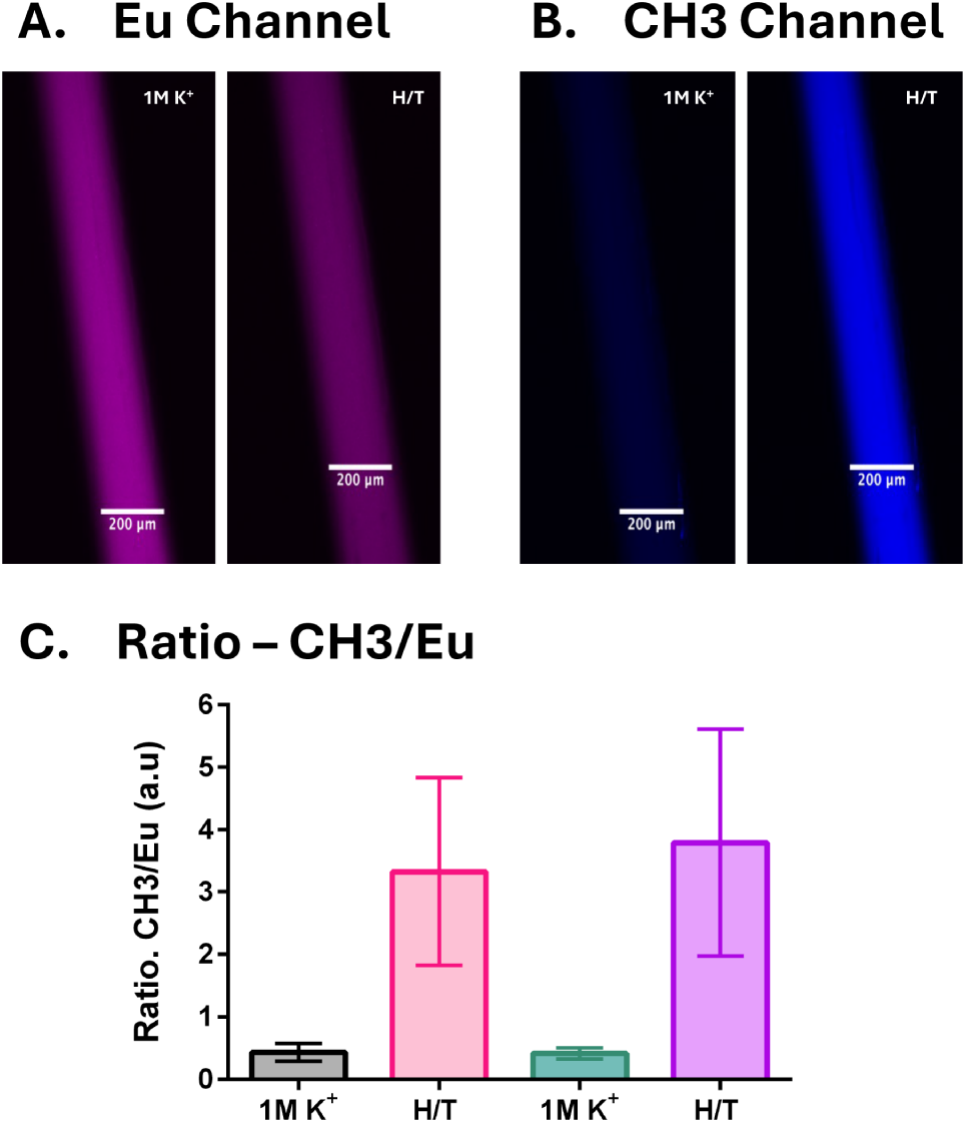
Reversibility testing of the potassium sensor with Eu­(DBM)_3_Phen as viewed on a confocal microscope (*n* = 3). (A) The Eu channel shows the response of the Eu-chelate in the nanosensor (Ex: 405 nm). The signal decreases when the analyte solution is changed from 1 M K^+^ to H/T buffer. (B) The CH3 channel shows the response of the CH3 in the nanosensor (Ex: 635 nm). The signal of the CH3 increases when the concentration of the analyte decreases (1 M K^+^ to H/T buffer), which is opposite to that of the Eu channel. (C) Ratiometric response (CH3/Eu) over two cycles of 1 M KCl and H/T exposure, showing a strong, reversible response to changing analyte. The error bar represents the variation in signal intensity between replicates.

We evaluated both the colloidal and functional stabilities of the Eu­(DBM)_3_Phen potassium sensors. Overall, the sensors demonstrated good colloidal stability over 5 days, with minimal changes in particle size and ζ-potential (Table S3). The functional stability showed a similar trend over 4 days. While there was a shift in the luminescence response over time, the ratiometric signals of the CH3/CH3 (λ_*em*_ = 680/585) and CH3/Eu (λ_*em*_ = 680/614) showed no significant changes between day 1 and day 4 (Figure S10). A clearer shift was observed by day 8, likely due to the Eu-chelate and CH3 signals degrading at different rates. Nonetheless, these results indicate that the sensor is stable over short time frames (*<*1 week), but more work needs to be done to increase its long-term stability. In addition to this, we compared the performance of the sodium sensors with that of Eu­(DBM)_3_Phen against a traditional sodium sensor. As seen in Figure S11, the sensor with the Eu-chelate seemed to have a lower sensitivity than its traditional counterpart, as quantified by their LogEC50 (−2.987 for the sodium sensor with Eu-chelate versus −3.464 for the traditional sodium nanosensor). However, this difference is not a significant limitation, as it can be bridged via formulation-based approaches. As highlighted by Dubach et al.,^80^ the sensitivity of ion-selective nanosensors can be modulated by adjusting the amount of ionophore within the sensing phase, leading to a more sensitive response.

Given that lanthanide complexes can be quenched through nonradiative pathways, we also tested the response of Eu­(DBM)_3_Phen to varying oxygen concentrations. Specifically, we cycled the oxygen levels from 0 mg/L to 4.46 mg/L (i.e., 0% to 21% O_2_ gas-phase equivalent) by alternating between N_2_ (0% O_2_) and air (21% O_2_) streams. The signal from Eu­(DBM)_3_Phen remained unchanged across oxygen concentrations, indicating that this complex is stable under ambient O_2_ conditions (see Figure S12).

While there is an ion-selective response, it is important to evaluate whether the signal arises from the gating effect or as a result of an analyte–Eu-chelate interaction. As highlighted by previous work,^41,43^ Eu-chelate emissions can be influenced by environmental changes, such as changes in ionic strength or pH. To probe this, we conducted control experiments using (i) Eu-only formulations (no ionophore or CH3), (ii) nanosensors without Eu, (iii) a sensor with an alternative chromoionophore (BB-dye), and (iv) an experiment to test reporter retention.

As shown in Figure 4C, Eu­(DBM)_3_Phen-only nanoparticles did not exhibit ion-selective behavior at low analyte concentrations, as expected. At higher levels (*>*10 mM), monovalent (Na^+^, K^+^) cations increased the luminescence, while divalent cations (Ca^2+^, Mg^2+^) decreased it. This behavior could be attributed to how these cations interact with the Eu-chelate inside the nanoparticle. It has been reported that Na^+^ and K^+^ can enhance Eu^3+^ luminescence by reducing nonradiative relaxation pathways via decreased hydration and increased structural compaction around the Eu^3+^ center, which improves energy transfer efficiency from ligand to metal.^81^

**Figure 4:**
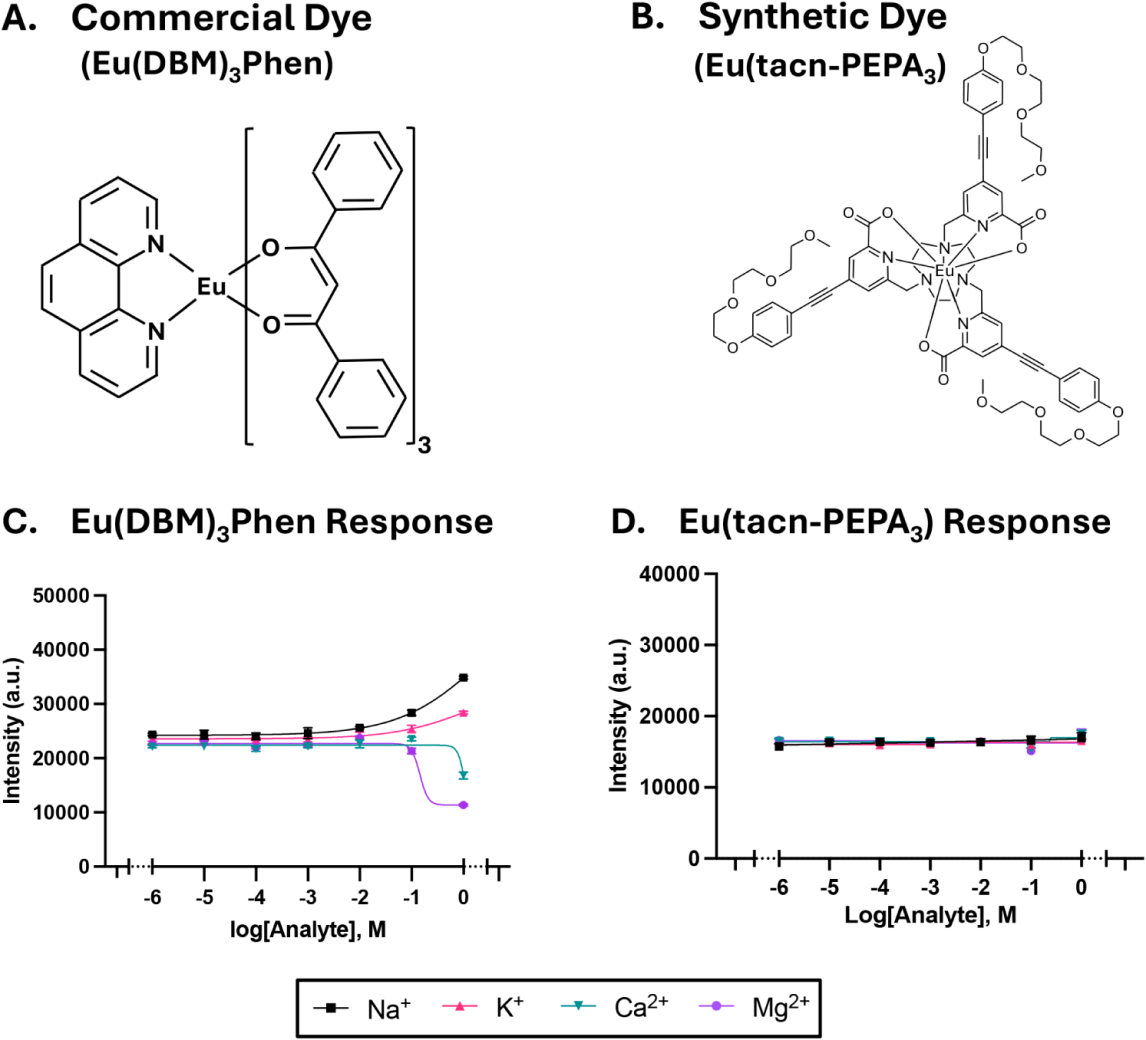
Response of the commercial and synthetic Eu-chelate to various biologically relevant cations (*n* = 3). (A,C) Eu­(DBM)_3_Phen, as previously described, is not affected by the various cations at low concentrations but at concentrations exceeding 10 mM, a decrease in signal is observed with divalent cations, while an increase is seen with its monovalent counterparts. (B,D) Chemical structure of Eu­(tacn-PEPA_3_), where the 1,4,7-triazacyclononane (tacn) and pegylated aryl alkyne (PEPA) stabilize the europium ion. Unlike its commercial counterpart, the Eu­(tacn-PEPA_3_)’s luminescence is not affected by either monovalent or divalent cations. Where they are not visible, error bars are smaller than the data points.

Divalent cations, on the other hand, play a different role. In their report, Kautenberger^82^ suggests that divalent cations like Ca^2+^ and Mg^2+^ impact solvation around the Eu-chelate and potentially displace it from the ligand.^82^ Additionally, when tested under nonbuffered conditions, such as acid/base conditions (pH 1.0 and pH 14.0, respectively), there was a noticeable shift in the luminescence of the Eu­(DBM)_3_Phen-only nanoparticles (Figures S13A and S14). In acidic conditions, there was almost no Eu signal, while under basic conditions, the signal was lower at pH 14.0 than at pH 12.0.

We also tested the ion-selective response in the absence of the Eu-chelate to confirm that the sensing response originates from CH3 and other sensing components rather than the Eu-chelate–ion interaction. As seen in Figure S15, we were able to obtain a potassium-selective response via the potassium sensors. This confirms that the sensing response originates from the sensors’ other components, as expected, rather than the direct Eu-chelate–chelateion interaction.

We also demonstrate that this ion-selective gating mechanism is not limited to CH3 and that any chromoionophore can be used, provided its absorbance changes at the Eu-chelate emission wavelength. In contrast to CH3 (whose absorbance between 600 and 700 nm decreases with increasing analyte), the absorbance of BB-dye increases across all wavelengths (Figure S16). As a result, we developed a sodium sensor using BB-dye, where the Eu-chelate emission decreases with rising analyte concentration, which matches the expected gating behavior and further affirms that absorbance modulation between 600 and 650 nm is sufficient to gate Eu emission, regardless of the chromoionophore used (Figure S17A).

Finally, we also tested the retention of the Eu-chelates within our sensors to evaluate whether the chelates were within the sensing phase or in the bulk solution. When compared against the stock nanosensor, the retentate had 75% and 82% of the Eu­(DBM)_3_Phen and CH3 (680 nm emission) signals, respectively, with no observable signal in the flow-through, indicating good dye retention (Figure S18). While the Eu-dye’s retention is lower than that of the stock nanosensor solution, this can be explained by some of the dye/nanoparticles being trapped within the Amicon filters, leading to lower signal output.

Additionally, we also fabricated other sensors with synthesized Eu-chelates—Eu­(tacn-PEPA_3_) and Eu­(DBM)_3_Bipy. Similar to their commercial counterpart, we fabricated sodium and potassium nanosensors, respectively, using CH3 to modulate luminescence. Both demonstrated ion-selective responses, with the former having a sodium-selective response, with a LogEC50 of −1.35, and a selectivity coefficient of −0.85 (see Table S2 in the Supporting Information for all selectivity values). The latter also demonstrated an ion-selective response (K^+^ with a LogEC50 of −3.98 and a selectivity coefficient of −3.26, which is comparable to those of the potassium sensors fabricated with Eu­(DBM)_3_Phen (Figure S19). It is worth noting that while the sodium sensors with Eu­(tacn-PEPA_3_) had a lower selectivity coefficient, this can be improved by modulating the ratio of the ionophore, charge balancer, and chromoionophore, as shown for ionophore-based sensors by others.^83^

In terms of their stability, we only monitored functional stability, not colloidal stability, as all the sensing materials apart from the Eu-chelate are the same. We noticed that Eu­(tacn-PEPA_3_) had a good ratiometric response for up to 4 days (Figure S20), despite the CH3 signal drifting over time. However, Eu­(DBM)_3_Bipy did not show a similar response. Within 24 h, the Eu signal had degraded significantly compared to CH3 (see Figure S21), suggesting that the dye might not have been fully incorporated into the sensing phase, which was further validated by retention tests. Figures S22 and S23 show the retention of Eu­(tacn-PEPA_3_) and Eu­(DBM)_3_Bipy, respectively. Compared to the stock, Eu­(tacn-PEPA_3_) and CH3 showed a retention of 82% and 99%, respectively. Whereas, Eu­(DBM)_3_Bipy showed 50% retention, which is further supported by the emission spectra (Figure S23B), where the CH3 signal almost overlaps, but the retentate and flow-through have half the signal of the stock solution. A possible explanation for the poor performance of Eu­(DBM)_3_Bipy could be due to poor lipophilicity of the complex. DBM has a calculated octanol–water partition coefficient (logP) of 2.2 (ChemDraw), while the Bipy ligand exhibits a lower logP of 1.88 (ChemDraw). This lower lipophilicity could explain the leaching of the Eu-chelate from the hydrophobic nanosensor matrix into the aqueous phase over time, resulting in poor retention values. Although further investigation is required to eliminate other confounding mechanisms, the comparative data for the three Eu-chelates, including luminescence lifetimes, pH stability, ionic interference resistance, and retention rates, is summarized in Table S4. As a result, we proceeded to further evaluate Eu­(tacn-PEPA_3_) for its lifetime, Eu-chelate–ion interaction, and function with an alternative chromoionophore.

To evaluate lifetime and ion interactions, we fabricated the Eu-chelate-only nanoparticle (no sensing components). The Eu­(tacn-PEPA_3_) had a lifetime of 549 μs, which is much longer than its commercial counterpart. Furthermore, it showed almost no sensitivity to various monovalent and divalent cations (Figure 4D) and only a small decrease in response to acidic solutions (pH 1.0) (Figure S13B). In short, this chelate is more robust in its cation-related interactions, but its signal can be impacted in low-pH systems. Finally, we also fabricated a sodium sensor using BB-dye and were able to obtain a sodium-selective response over potassium (see Figure S17B), indicating that the chromoionophore is gating the analyte emission and the Eu­(tacn-PEPA_3_) by itself is not analyte sensitive.

While the sensors we developed in this work show promise, there is room for further improvement. First, as seen in the Eu-chelate-only tests, the complexes are sensitive to pH variances. Kokko et al.^90^ demonstrate how polystyrene shells can be used to protect the chelate from pH effects. However, this has the caveat of the particle being rather large, which can make encapsulation into nanosensors difficult. Another approach would be to use structures like the picolinate-based coordination polymers or MOFs, as highlighted by Jornet-Molla et al.^84^ and Wang et al.,^85^ both of which showed pH stability from pH 4 to pH 11. Another area of improvement would be to make these structures more compatible with bioimaging. Currently, while these lanthanides have large energy separation between excitation and emission wavelengths, they are often excited in the UV region, posing a significant challenge for bioimaging. In this spectral range, the excitation light is prone to scattering and absorption by endogenous chromophores (e.g., hemoglobin, melanin), which substantially reduces signal-to-noise ratios,^86^ limiting the effective penetration depth to only a few millimeters. These interactions not only restrict imaging depth but also increase the risk of photodegradation and interference from background autofluorescence. A possible solution could be to develop chelating structures that exhibit absorption profiles at higher wavelengths or to use upconverting nanostructures, as demonstrated by Wolfbeis’ group.^87^ Finally, further development of NIR lanthanide complexes offers a promising route toward analyte profiling in complex biological systems, benefiting from deep tissue penetration and reduced background noise.^88,89^ To bypass the limitations of UV excitation, recent strategies have focused on coordination sphere engineering to red-shift emission. For instance, Zheng and coworkers^91^ developed a NIR-emitting Ce­(III) complex. They utilized sulfur-coordinating ligands, such as dithiobiurets, instead of traditional oxygen/nitrogen donors to sulfur-coordinating ligands to significantly red-shift the emission. However, as highlighted by Zhu et al.^88^ in their review, maintaining high quantum yields in the NIR-II window remains a challenge. The authors highlight that ligand fluorination could be used as a tool to enhance the shielding effect of the ligand and enhance quantum yields. Furthermore, while these signals decay over time, their long luminescence lifetimes are a distinct advantage, enabling time-gated detection to eliminate short-lived background autofluorescence and improve imaging resolution. Cosby et al. provide a more in-depth coverage of NIR lanthanides for bioimaging applications.^89^

## Conclusions

In this work, we demonstrate how europium (Eu)-chelates can be employed in a modular approach to ion detection through sensor fabrication. Using flash nanoprecipitation, we developed sodium- and potassium-selective sensors incorporating both commercially available and synthetically derived Eu-chelates, specifically Eu­(DBM)_3_Phen and Eu­(tacn-PEPA_3_), respectively.

These sensors exhibited reversible ion-selective responses to their target ions, even in the presence of biologically relevant competing ions. Notably, the commercially available Eu­(DBM)_3_Phen sensormaintained performance across a broad oxygen range (0% to 21%), highlighting its robustness under varying physiological conditions. To validate the modularity of our sensing platform, we replaced the chromoionophore with an alternative dye (BB-dye) in the sodium sensor. The resulting performance confirmed that the gating mechanism is dependent on the choice of chromoionophore, reinforcing the modular design principle. While Eu­(DBM)_3_Phen performed well, it exhibited a relatively short luminescence lifetime (131 μs) and showed sensitivity to pH fluctuations and interference from divalent cations at elevated concentrations. In contrast, the synthetic Eu­(tacn-PEPA_3_) complex offered several advantages: it had a longer lifetime (549 μs), reduced sensitivity to both monovalent and divalent ionic interference, greater pH stability, and superior retention (82% Eu, 99% CH3). However, these benefits come with the trade-off of requiring in-house synthesis, as opposed to the convenience of a commercially available alternative.

Nonetheless, both chelates demonstrate a strong potential as next-generation reporters for biocompatible, time-gated ion sensing. Future efforts will focus on enhancing chelate stability, minimizing pH sensitivity through improved encapsulation strategies, and red-shifting excitation wavelengths to reduce phototoxicity and enhance biocompatibility. Additionally, developing NIR-II lanthanide complexes will be crucial for extending these sensors to broader in vivo and imaging applications.

## Supplementary Material



## Notes

Table S1, Nanosensor optode formulation, components, and values; Table S2, Midpoint response and selectivity coefficients of Na^+^ and K^+^ sensors; Table S3 , Particle size and zeta-potential of K^+^ sensors over time; Table S4, Comparative analysis of the Europium chelates; Table S5, One-phase decay fit parameters and values for the emission profiles; Figure S1, Chromatographic analysis of Eu­(tacn–PEPA_3_) and Eu­(DBM)_3_Bipy; Figure S2, ESI of Eu­(DBM)_3_Bipy and Eu­(tacn–PEPA_3_); Figure S3, Response of Na^+^ sensors containing Eu­(DBM)_3_Phen to cationic analytes; Figure S4, Response of Na^+^ sensors containing Eu­(DBM)_3_Phen to various anions; Figure S5, Absorbance profile of CH3 in K^+^ sensors; Figure S6, Emissive lifetime of Eu­(tacn–PEPA_3_) and Eu­(DBM)_3_Phen; Figure S7, Emission profile of CH3 with a delay of 150 μs; Figure S8, Reversibility data of Eu-chelate and CH3 in potassium nanosensors; Figure S9, Impact of temperature on Eu­(DBM)_3_Phen emission; Figure S10, Functional stability of potassium nanosensors containing Eu­(DBM)_3_Phen; Figure S11, Normalized comparison of Eu-chelate Na^+^ sensors vs traditional Na^+^ sensors; Figure S12, Impact of dissolved O_2_ on Eu­(DBM)_3_Phen emission; Figure S13, Impact of various cations on Eu-chelates emission; Figure S14, Impact of pH on Eu­(DBM)_3_Phen emission; Figure S15, Potassium sensors’ response to analyte; Figure S16, BB-dye absorbance profile as a function of analyte concentration; Figure S17, Na^+^ sensors response to analyte (containing Eu-chelate and BB-dye); Figure S18, Dye retention testing of K^+^ sensors fabricated with Eu­(DBM)_3_Phen; Figure S19, Response of K^+^ sensors containing Eu­(DBM)_3_Bipy to analyte; Figure S20 , Functional stability of Na^+^ nanosensors containing Eu­(tacn–PEPA_3_); Figure S21, Temporal response of sodium sensors containing Eu­(DBM)_3_Bipy; Figure S22, Dye retention testing of Na^+^ sensors containing Eu­(tacn–PEPA_3_); Figure S23, Dye retention testing of Na^+^ sensors containing Eu­(DBM)_3_Bipy (PDF)

## Abbreviations

CH3: chromoionophore 3
Tacn: 1,4,7-triazacyclononane
PEPA: pegylated aryl alkyne
Eu,: europium
DBM: dibenzoylmethane
Phen: phenanthroline
Bipy: bipyridine
H/T: Hepes-tris
NIR: near-infrared
BB-dye: blueberry dye
ISE: ion-selective electrode
