# Supplementary material for: Modular Europium-Based Nanosensors for the Detection of Sodium and Potassium Ions
Source: ACS Omega. 2026 Apr 20;11(17):25712–23. doi: 10.1021/acsomega.6c00549 (PMC13150572; doi:10.1021/acsomega.6c00549)
Supplement: Supplementary file 1 [file ao6c00549_si_001.pdf]

Supporting Information

Modular Europium-Based Nanosensors for The Detection of Sodium and Potassium Ions

Adrian A. Mendonsa [1], Cameron L. Lyman [2], Logan C. Ruthardt [1], Raphael Lengacher [3], M. Andrey Joaqui-Joaqui [3], Mohammad Al Mesfer [4], Matt J. Kipper [4], Eszter Boros [3] and Kevin J. Cash [1,2*]

1) Department of Chemical and Biological Engineering. Colorado School of Mines, Golden, CO 80401, United States of America
2) Quantitative Bioscience and Engineering Program. Colorado School of Mines, Golden, CO 80401, United States of America
3) Department of Chemistry. University of Wisconsin-Madison, Madison, WI 53706, United States of America
4) School of Biomedical and Chemical Engineering. Colorado State University, Fort Collins, CO 80523, United States of America

Table S1	Nanosensor optode formulation, components and values
Table S2	Midpoint response and selectivity coefficients of Na^+ and K^+ sensors
Table S3	Particle size and zeta-potential of K^+ sensors over time
Table S4	Comparative analysis of the Europium chelates
Figure S1	Chromatographic analysis of Eu(tacn-$PEPA_3$)
Figure S2	ESI of Eu$(DBM)_3$Bipy and Eu(tacn-$PEPA_3$)
Figure S3	Response of Na^+ sensors containing Eu$(DBM)_3$Phen to cationic analytes
Figure S4	Response of Na^+ sensors containing Eu$(DBM)_3$Phen to various anions
Figure S5	Absorbance profile of CH3 in K^+ sensors
Figure S6	Emissive lifetime of Eu(tacn-$PEPA_3$) and Eu$(DBM)_3$Phen
Table S5	One phase decay fit parameters and values for the emission profiles
Figure S7	Emission profile of the CH3 with a delay of 150 μs
Figure S8	Reversibility data of Eu-chelate and CH3 in potassium nanosensors
Figure S9	Impact of temperature on Eu$(DBM)_3$Phen emission
Figure S10	Functional stability of potassium nanosensors containing Eu$(DBM)_3$Phen
Figure S11	Normalized comparison of Eu-chelate Na^+ sensors vs traditional Na^+ sensor
Figure S12	Impact of dissolved O_2 on Eu$(DBM)_3$Phen emission
Figure S13	Impact of various cations on Eu-chelates emission
Figure S14	Impact of pH on Eu$(DBM)_3$Phen emission
Figure S15	Potassium sensors only response to analyte

Figure S16 BB-dye absorbance profile as a function of analyte concentration
Figure S17 Na sensors response to analyte (containing Eu-chelate and BB-dye)
Figure S18 Dye retention testing of K^+ sensors fabricated with $Eu(DBM)_3Phen$
Figure S19 Response of K^+ sensors containing $Eu(DBM)_3Bipy$ to analyte
Figure S20 Functional stability of Na^+ nanosensors containing Eu(tacn-$PEPA_3$)
Figure S21 Temporal response of sodium sensors containing $Eu(DBM)_3Bipy$
Figure S22 Dye retention testing of Na^+ sensors containing Eu(tacn-$PEPA_3$)
Figure S23 Dye retention testing of Na^+ sensors containing $Eu(DBM)_3Bipy$

Table S 1: Nanosensor optode formulation, components and values.

**Optode Components**	**Sodium Sensor**	**Potassium Sensor**	**(BBDye) Sodium Sensor**
PS-PEG	2.50 mg	2.50 mg	2.50 mg
Polystyrene	0.50 mg	0.50 mg	0.50 mg
BEHS	2.4 µL	2.4 µL	2.4 µL
Eu dye (varies by dye)	0.5 mg for Eu(DBM)$_3$Phen and 0.05 mg for Eu(tacn-PEPA$_3$)		
NaBARF	0.15 mg	0.25 mg	0.50 mg
CH 3	0.025 mg	0.0625 mg	
BB Dye	-	-	0.25 mg
NaI X	0.75 mg	-	0.75 mg
KI-I	-	0.375	-
Quench Bath (H/T buffer)	4.0 mL	4.0 mL	4.0 mL

Table S 2: Midpoint response (LogEC50) and selectivity coefficients (LogK($_{i,x}$)) of the sodium and potassium sensors to target and competing analytes.

**Analyte**	**Sodium Sensor**		**Potassium Sensor**	
	**LogEC50**	**LogK($_{Na,x}$)**	**LogEC50**	**LogK($_{K,x}$)**
**Na^+**	-1.80	----	0.054	-3.31
**K^+**	-0.19	-1.61	-3.26	----
**Ca^{2+}**	>10	>-10	>10	>-10

Table S 3: Particle size and zeta-potential values for potassium sensors containing $Eu(DBM)_3Phen$ evaluated over 4 days.

		**Day 1**	**Day 2**	**Day 4**
**DLS**	Size (nm)	103 $\pm$ 3.04	122 $\pm$ 4.47	126 $\pm$ 0.93
	Polydispersity	0.172 $\pm$ 0.054	0.124 $\pm$ 0.030	0.118 $\pm$ 0.038
**ζ - potential**	Zeta Pot. (mV)	-18.13 $\pm$ 2.08	-20.09 $\pm$ 1.06	-18.61 $\pm$ 1.20
	Mobility (μ/s)/(V/cm)	-1.42 $\pm$ 0.16	-1.57 $\pm$ 0.08	-1.45 $\pm$ 0.09

Table S 4: Comparative analysis of the Europium chelates. A summary of their luminescent lifetime, pH Stability, resistance to ionic interference and retention rates.

	$Eu(DBM)_3Phen$	$Eu(tacn\text{-}PEPA_3)$	$Eu(DBM)_3Bipy$
Luminescent lifetime	549 μs	131 μs	Not characterized
Ph stability within nanosensor	Stable at pH 7, signal impacted at pH >4 and <12	Stable at pH 7 - 13, signal impacted at pH >4	pH stability not characterized
Resistance to ionic interference within nanosensor	Not impacted by Na, K, Ca, and Mg between 1μM – 10 mM concentration, changes in signal seen for Ca^{2+} and Mg^{2+} conc. >0.1 M. Signal decreases significantly in Fe^{3+} and Zn^{2+} solutions > 1mM	Not impacted by Na, K, Ca and Mg between 1μM – 1M concentration	Sensitivity response of the complex by itself not characterized
Retention rates within nanosensor (Retentate compared to stock)	71.4%	81.3 %	49.1%

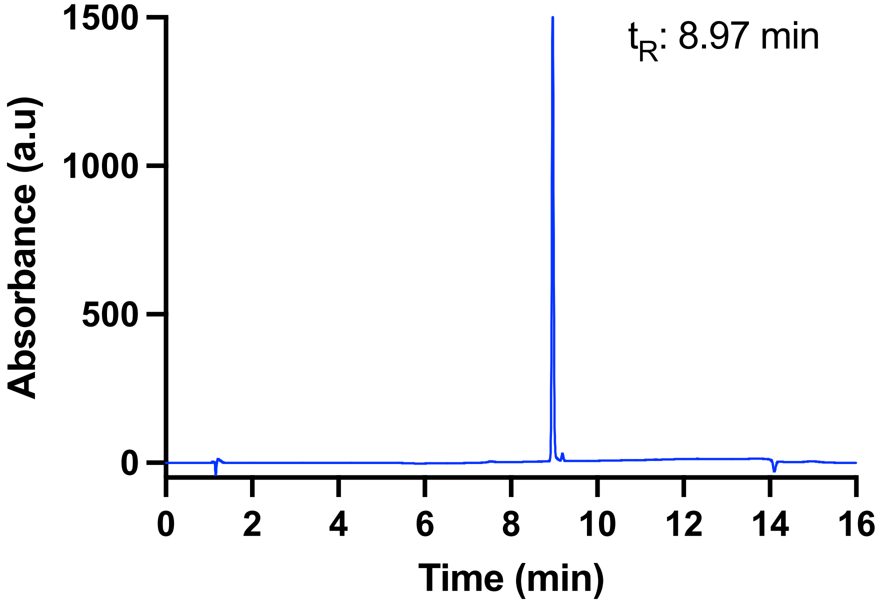

Figure S 1: Chromatographic analysis of Eu(tacn-PEPA$_3$). Absorbance monitored at 254 nm. Retention time (t_R) = 8.97 min LCMS analysis was carried out on a Phenomenex Luna® C18 column (5 µm, 150 mm × 3 mm, 100 Å) at a flow rate of 0.8 mL/min using a single quadrupole Agilent 1200 Infinity II LC/MSD system equipped with a binary gradient pump, UV-vis detector, automatic injector, and an atmospheric pressure electrospray ionization (AP-ESI) source. Ultraviolet absorption was recorded at 220 nm and 254 nm, and positive and negative mass spectra were collected from m/z = 100-2000. Method A: Gradient: 0-3 min: 5% B; 3-10 min: 5-95% B; 10-12 min: 95% B; 12-12.5 min: 95-5%; B; 12.5-16 min: 5%. A = water + 0.1% aqueous formic acid; B = MeCN + 0.1% formic acid.

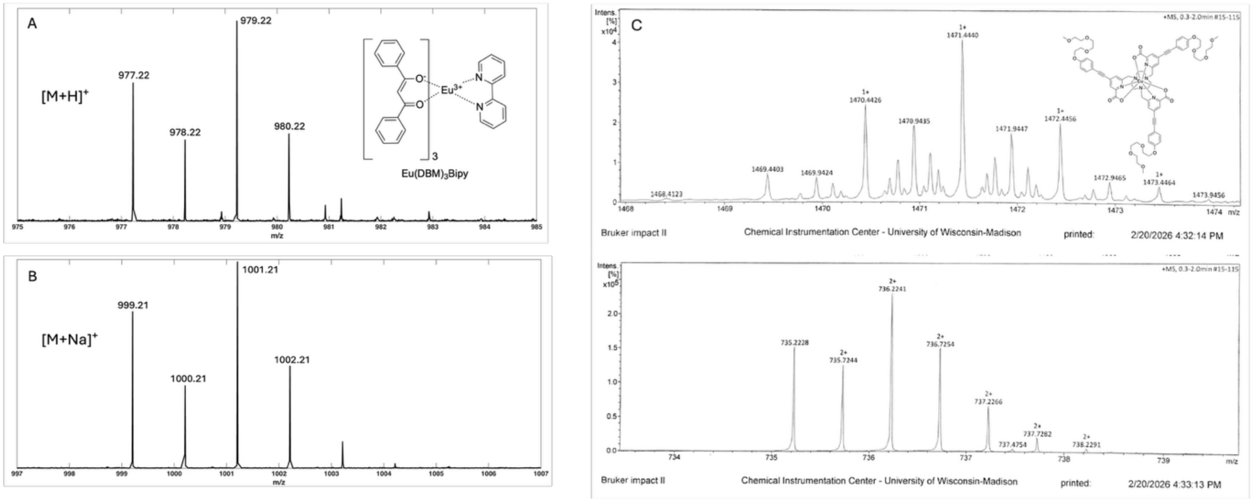

Figure S 2: ESI of Eu(DBM)$_3$Bipy and ESI-HRMS of Eu(tacn-PEPA$_3$). (A) Proton adduct [M+H]$^+$. Calc m/z for $C_{55}H_{41}EuN_2O_6$ [M+H]$^+$: 977.2236 (^{151}Eu) and 979.2250 (^{153}Eu). Observed: 977.22, 979.22, with M+1 isotopic peaks at 978.22 and 980.22. (B) Sodium adduct [M+Na]$^+$. Calc m/z for $C_{55}H_{41}EuN_2O_6$ [M+Na]$^+$: 999.2055 (^{151}Eu) and 1001.2069 (^{153}Eu). Observed: 999.21, 1001.21, with M+1 isotopic peaks at 1000.21 and 1002.21. To characterize the Eu(DBM)$_3$Bipy , the sample (50 μM in ethanol) was introduced by direct infusion at 20 μL/min. Source parameters were adjusted to promote soft ionization conditions (capillary 2.5 kV, cone 30 V, source temperature 50 °C, desolvation temperature 40 °C). Mass spectra were collected over m/z 950–1050. Electrospray ionization mass spectrometry (ESI-MS) was performed at the Colorado State University Analytical Resources Core (ARC) using a Waters Synapt G2-Si QTOF mass spectrometer operating in positive ion mode (C) Calc m/z for $C_{72}H_{82}EuN_6O_{18}$ [M+H]$^+$: 1471.4898. Observed: 1471.4440. Calc m/z for $C_{72}H_{83}EuN_6O_{18}$ [M+2H]$^{2+}$: 736.2488. Observed: 736.2241. This data was acquired via direct injection into a Thermo Scientific Q Exactive Focus Orbitrap MS system at the University of Wisconsin-Madison Department of Chemistry Paul Bender Chemical Instrumentation Center (CIC).

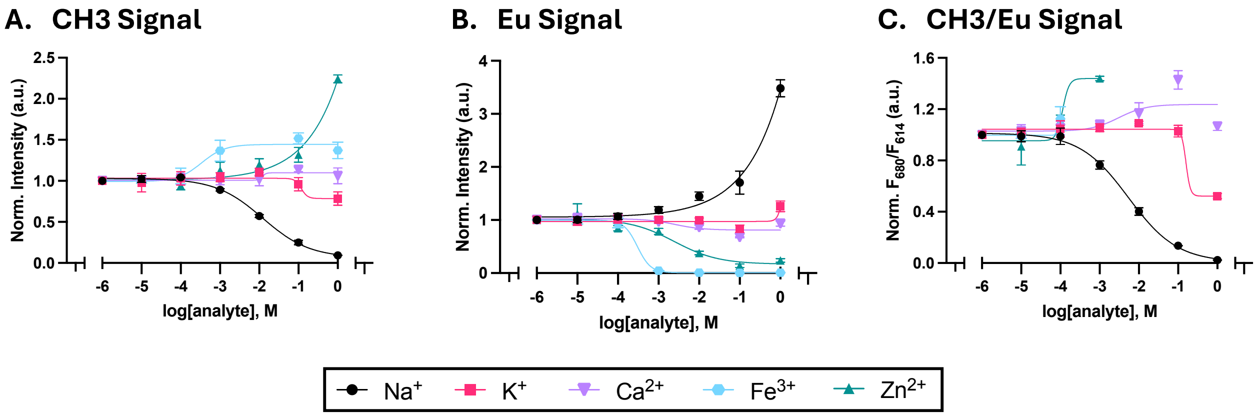

Figure S 3: Response of sodium sensors containing Eu(DBM)₃Phen to various cationic analytes (n=3). (A) The CH3 emission at 680 nm (Ex: 650 nm), shows an affinity towards Na^{+} ions as expected, however, the increase in signal for Fe^{3+} and Zn^{2+} at higher concentrations (> 1mM) indicates the CH3 is protonated due to shifts in the pH (B) Europium emission intensity. While the signal increases with [Na^{+}], the observed quenching in the presence of and indicates structural interference with the europium chelate. (C) Ratiometric response (CH3/Eu), illustrating the cumulative impact of cation interference. Data points above 1mM for Fe^{3+} and Zn^{2+} are omitted for visual clarity, due to the normalized signal exceeding 3.0 (a.u.). Error bars not visible are smaller than the data points.

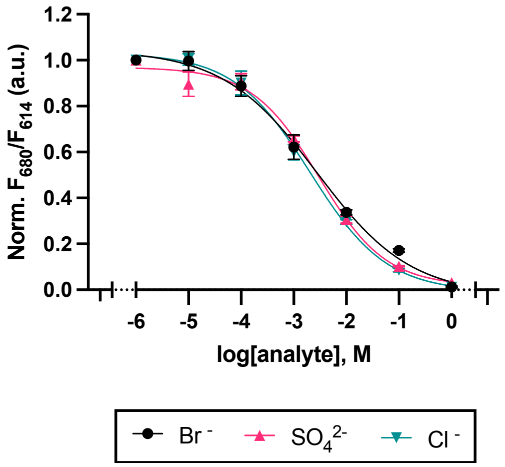

Figure S 4: Ratiometric response of sodium sensors containing Eu(DBM)₃Phen to sodium in the presence of various anions (n=3). The normalized ratiometric response (CH3/Eu) remains consistent across different sodium salts, regardless of the counterion (Br^{-},SO_4^{2-} or Cl^{-}). The overlapping sigmoidal profiles highlight that the sensor response is governed strictly by concentration of Na^{+} and is not significantly influenced by the anionic species present in the sample. Where not visible error bars are smaller than that of the data point.

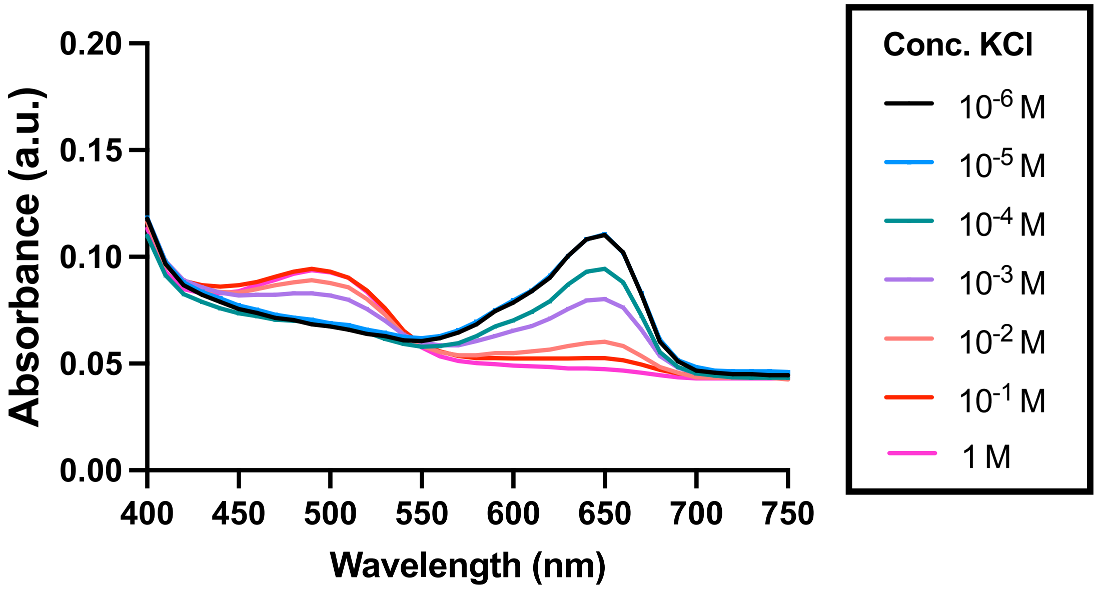

Figure S 5: Absorbance profile of the CH3 in potassium sensors containing Eu(DBM)$_3$Phen. The absorbance profile changes as a function of analyte concentration (n=3).

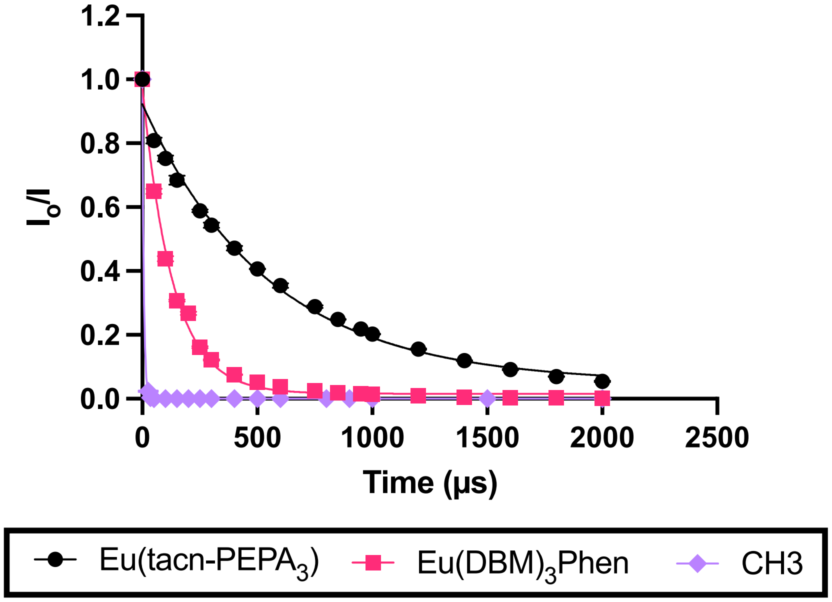

Figure S 6: Emissive lifetime of the Eu(tacn-PEPA$_3$) and Eu(DBM)$_3$Phen and CH3 collected in H/T buffer (n=3). The Eu-based sensors were fabricated without sensing components, just the Eu-chelates and the charge balancer. The CH3 sensor had the charge balancer and a sodium ionophore only, no Eu-chelates. All sensors were added in equal parts volume to the H/T buffer prior to data collection. The emission profile was obtained on the plate reader, with the delayed occurring from 0 μs – 2000 μs delay. The gain was adjusted to 80 for the Eu(DBM)$_3$Phen,120 for the Eu(tacn-PEPA$_3$) and 100 for the CH3 (680 nm emission). The data was fit to a one-phase decay model, the parameters can be seen below in Table S4.

Table S 5: One phase decay fit parameters and values for the emission profiles seen in Figure S6.

	Eu(tacn-PEPA$_3$)	Eu(DBM)$_3$Phen	CH3
One phase decay			
Y0	0.9224	0.9787	1.000
Plateau	0.04980	0.01577	-5.716×10^{-5}
K	0.001819	0.007630	0.1513
Half Life	381.0	90.84	4.582
Tau	549.7	131.1	6.611
Span	0.8726	0.9629	1.000

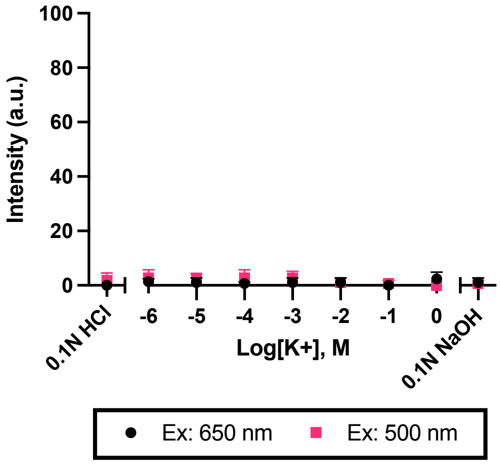

Figure S 7: Emission profile of the CH3 with a delay of 150 μs (n=3). No signal was observed from the CH3 dye.

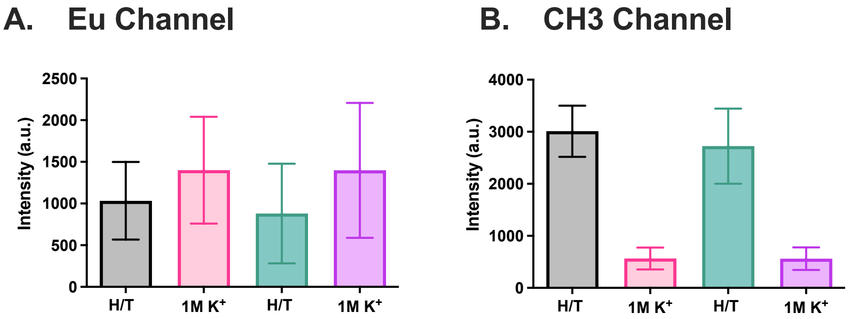

Figure S 8: Reversibility data of individual channels in potassium nanosensors containing Eu(DBM)$_3$Phen and CH3 (n = 3). (A) The Eu channel shows an overall reversible response to changes in analyte concentration over two cycles. However, relatively large error bars indicate signal variability, which is attributed to temperature fluctuations in the confocal microscope during measurement, conditions known to affect the Eu signal. (B) In contrast, the CH$_3$-only channel displays a clear and reproducible signal over two cycles, with smaller error bars. This increased stability is likely due to the CH$_3$ signal being less sensitive to temperature variations compared to the Eu channel.

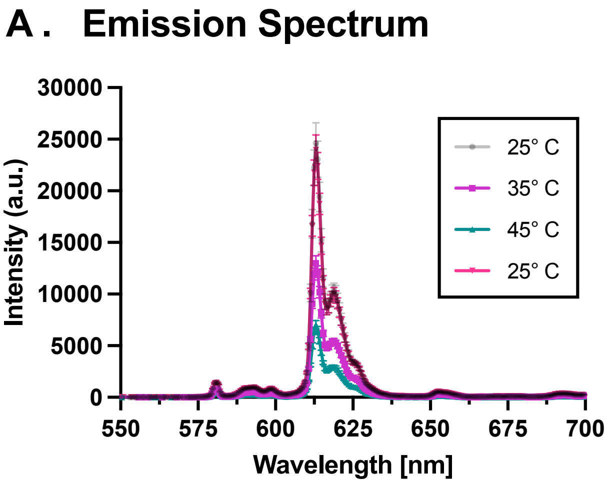

Figure S 9: Impact of temperature on the response of Eu(DBM)$_3$Phen (n = 3). To obtain this data, sensors were fabricated without additional sensing components, only the Eu-chelate and charge balancer were included. The sensors were loaded into quartz cuvettes and submerged in a temperature-controlled bath. At each temperature point, samples were equilibrated for 30 minutes before measurements were taken. Data were collected immediately after removal from the bath to minimize temperature-induced changes. (A) Emission spectra of the sensor at each temperature point were recorded using an Avantes spectrometer (λ_{ex}= 365 nm, slit width = 50 µm). As temperature increases, the Eu-chelate emission decreases; however, the signal fully recovers when the temperature returns to 25 °C. (B) Endpoint emission data, averaged at 614 nm from the emission spectra, provides a clearer view of the Eu-chelate signal recovery upon returning to baseline temperature.

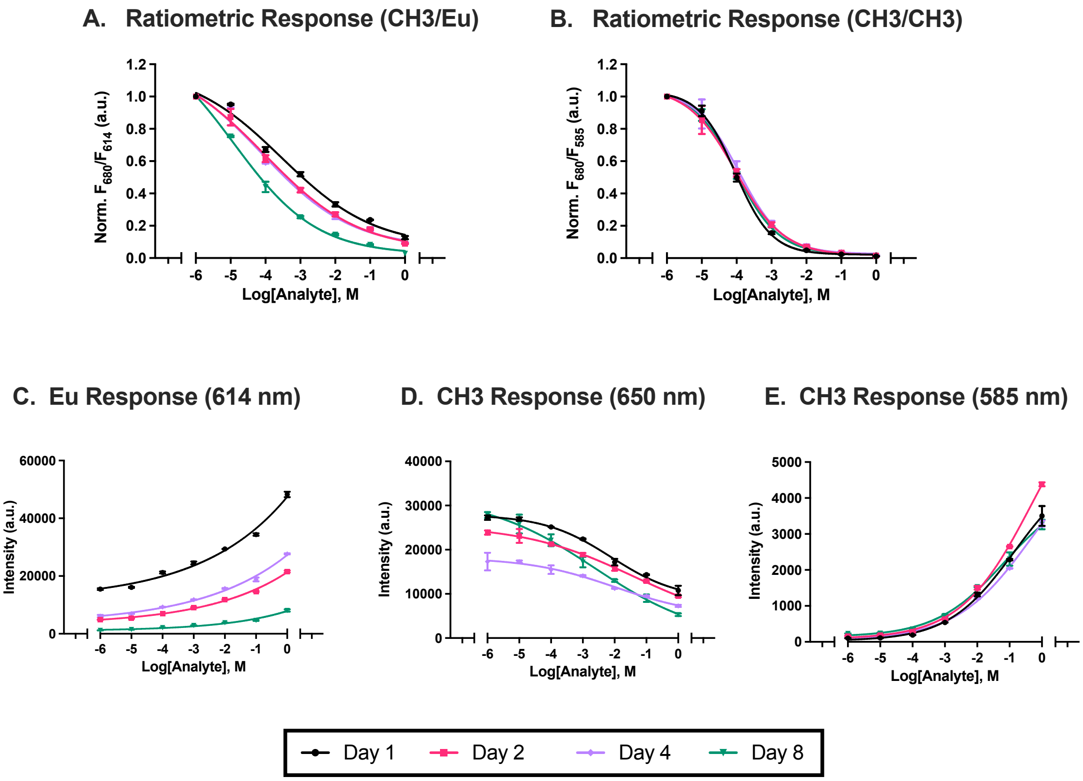

Figure S 10: Functional stability of potassium nanosensors containing $Eu(DBM)_3Phen$ over 8 days (n = 3), profiled against potassium analyte. (A) The normalized ratiometric signal (CH3/Eu, 680 nm / 614 nm) demonstrates good stability for up to 4 days. By day 8, a shift in the signal is observed, which can be attributed to changes in the responses of both the Eu-chelate and CH3 components, as shown in panels (C), (D), and (E). (B) The normalized ratiometric signal (CH3/CH3, 680 nm / 585 nm) remains stable over the full 8-day period. This stability is likely due to the intrinsically ratiometric nature of the CH3 signals, where a decrease in the 680 nm signal corresponds with an increase at 585 nm, supported by the trends observed in panels (D) and (E). (C) Emission profile of the Eu-chelate (λ_{ex} = 352 nm) over 8 days, showing a clear decline in signal intensity over time. (D) Emission profile of CH3 (λ_{ex}= 650 nm) over 8 days, also displaying a decreasing trend. (E) Emission profile of CH3 (λ_{ex} = 500 nm) over 8 days, where a slight increase in signal is observed. Where not visible, error bars are smaller than the data points.

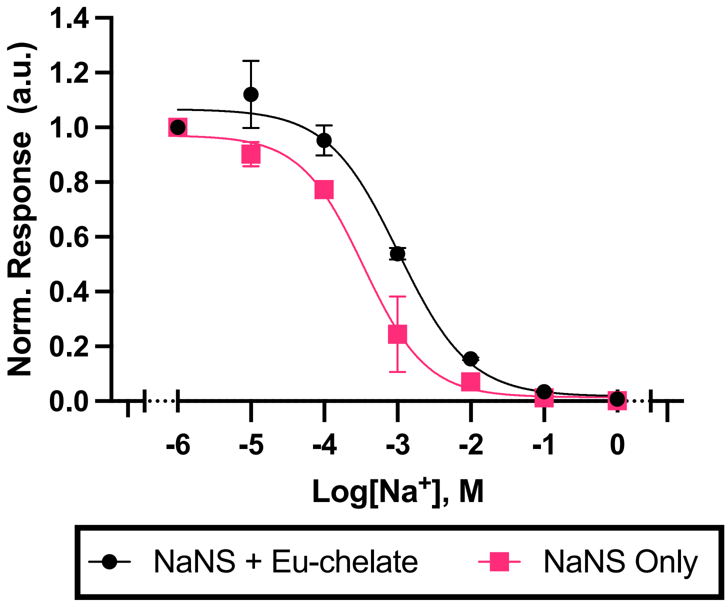

Figure S 11: Normalize response of sodium nanosensor (NaNS) with Eu(DBM)$_3$Phen compared against a traditional sodium nanosensor (NaNS) (n=3). The NaNS with the Eu-chelate (black circle, F_{680nm}/F_{614nm}) had a less sensitive response than its traditional counterpart (pink square, F_{680nm}/F_{585nm}) when profiled against Na^+ analyte as suggested by the LogEC50 (-2.987 NaNS + Eu-chelate and -3.464 for the NaNS only).Where not visible the error bars are smaller than that of the data point.

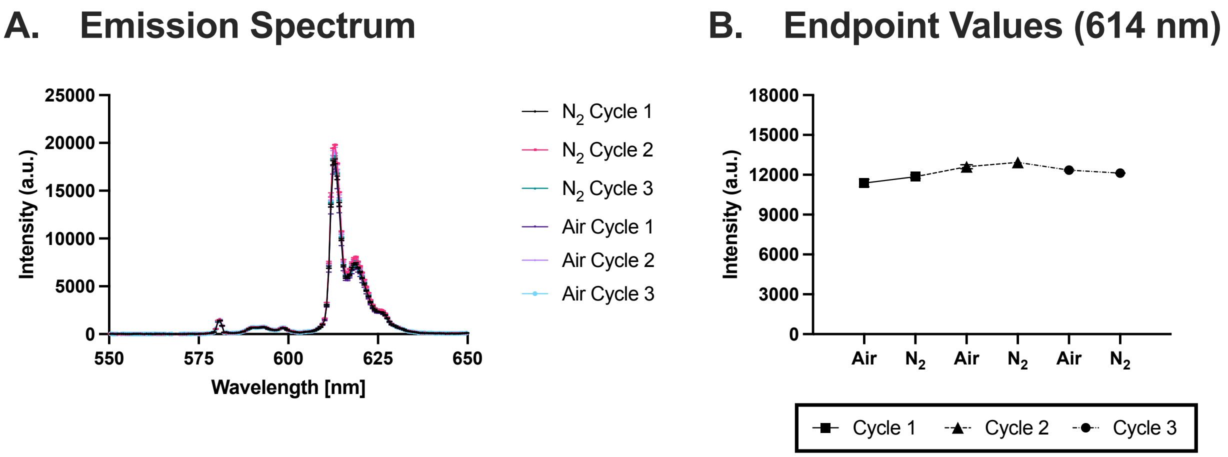

Figure S 12: Impact of varying O_2 concentration on the response of Eu(DBM)$_3$Phen (n = 3). To obtain this data, sensors were fabricated without additional sensing components—only the Eu-chelate and charge balancer were included. The sensors were loaded into quartz cuvettes and bubbled with gas mixtures according to the procedure described in the Methods section. (A) Emission spectra of the sensor at 0% and 21% dissolved O_2 (achieved using pure N_2 and air, respectively). No notable change in signal intensity was observed. (B) Endpoint emission data averaged at 614 nm from the spectra. While minor variations are present across cycles, they are not statistically significant. Data was obtained using an Avantes spectrometer (slit width = 50 μm) with excitation provided by a 365 nm LED light source. Where not visible, error bars are smaller than the data points.

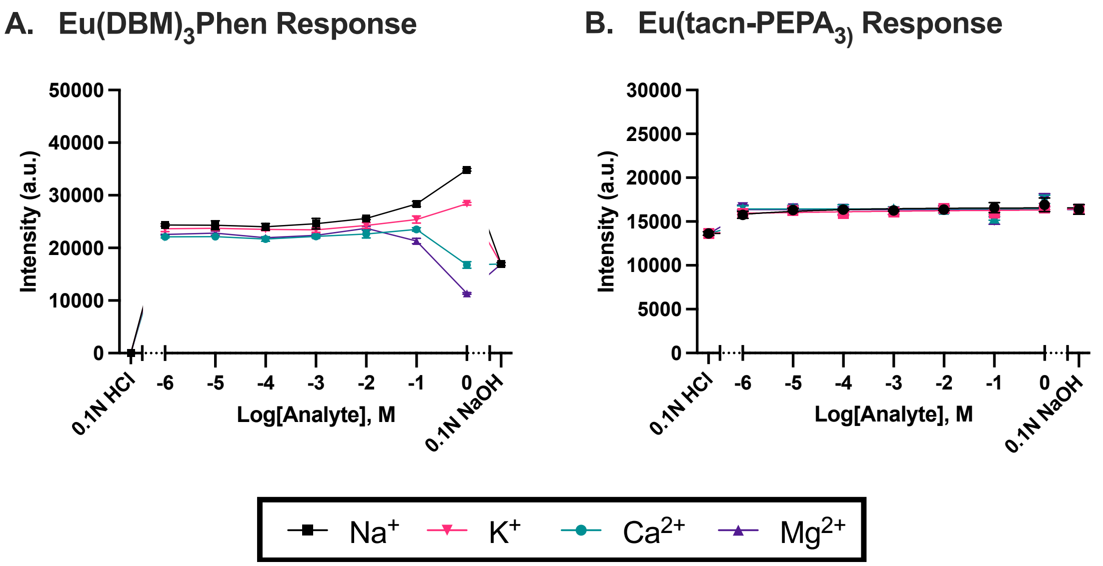

Figure S 13: Impact of various cations and pH on the response of Eu-chelates (n = 3). (A) The Eu(DBM)$_3$Phen chelate shows minimal response to cationic analytes at lower concentrations but exhibits a clear shift in signal at higher concentrations (>10 mM), as well as under extreme pH conditions (pH 1.0 and pH 14.0). This suggests that the complex may not be stable in unbuffered environments or systems with high ionic strength. (B) The Eu(tacn-PEPA$_3$) chelate shows little to no response to changing analyte concentrations or basic conditions (pH 14.0). However, a small decrease in signal is observed under acidic conditions (pH 1.0), indicating sensitivity to low pH while maintaining stability in high-ionic-strength systems. To obtain this data, sensors were fabricated without additional sensing components. Only the Eu-chelate and charge balancer were included. Both samples were excited at 352 nm. Emission was monitored at 614 nm for Eu(DBM)$_3$Phen and 620 nm for Eu(tacn-PEPA$_3$). Where not visible, error bars are smaller than the data points.

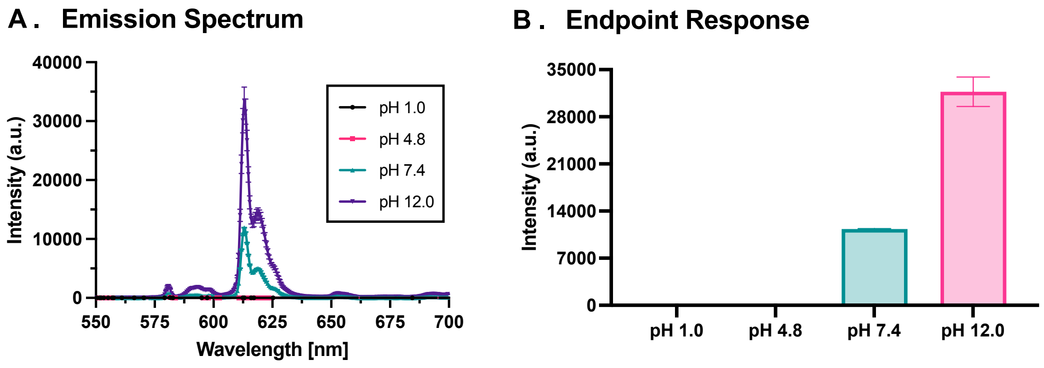

Figure S 14: Impact of pH on the response of Eu(DBM)$_3$Phen (n = 3). (A) As the pH of the surrounding solution increases from 1.0 to 12.0, the emission intensity of the Eu-chelate increases accordingly. (B) Endpoint emission data was obtained by averaging the intensity at 614 nm. To obtain this data, sensors were fabricated without additional sensing components; only the Eu-chelate and charge balancer were included. Samples were prepared in water, with 200 µL of the sensor solution added to 1800 µL of analyte to minimize any shift in final pH. The sample was excited at 365 nm, and emission from Eu(DBM)$_3$Phen was recorded at 614 nm using a 50 µm slit width. Where not visible, error bars are smaller than the data points.

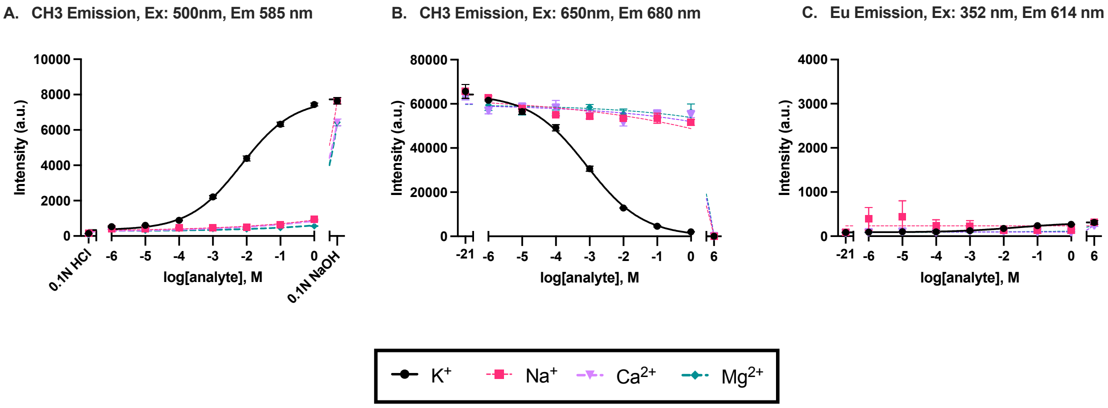

Figure S 15: *Response of a potassium nanosensor to various analytes in the absence of Eu(DBM)$_3$Phen (n = 3).* (A) As analyte concentration increases, the CH3 emission at 585 nm increases only in response to potassium, while remaining constant for all other ions. (B) The CH3 emission at 680 nm shows an inverse response, decreasing only in the presence of potassium. No significant change is observed for other ions. (C) No signal is detected in the Eu-channel, confirming that any Eu-related response observed in other experiments originates from the lanthanide chelate itself and not from CH3. Where not visible, error bars are smaller than the data points.

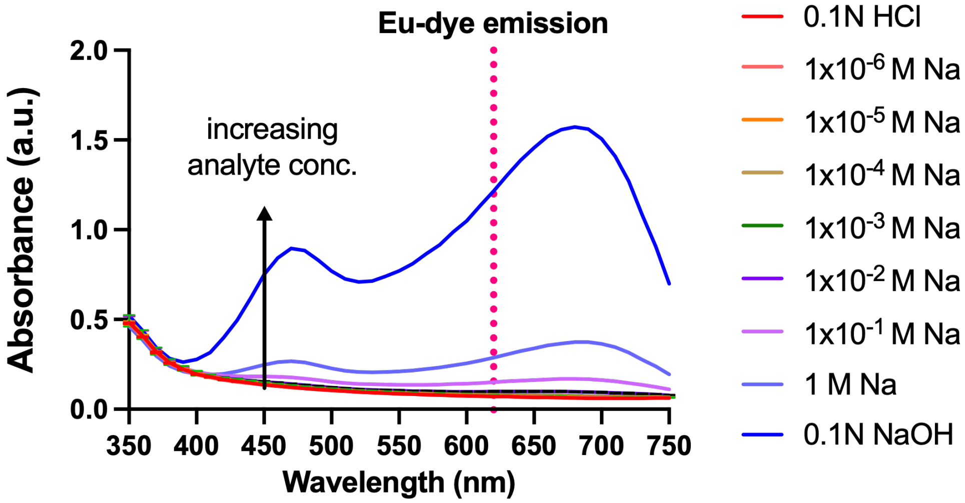

Figure S 16: Change in the absorbance of the BB-dye as a function of sodium concentration (n=3). As the concentration of the analyte increases, the BBy-dye deprotonates as a result, the absorbance of the dye increases between 400– 800 nm.

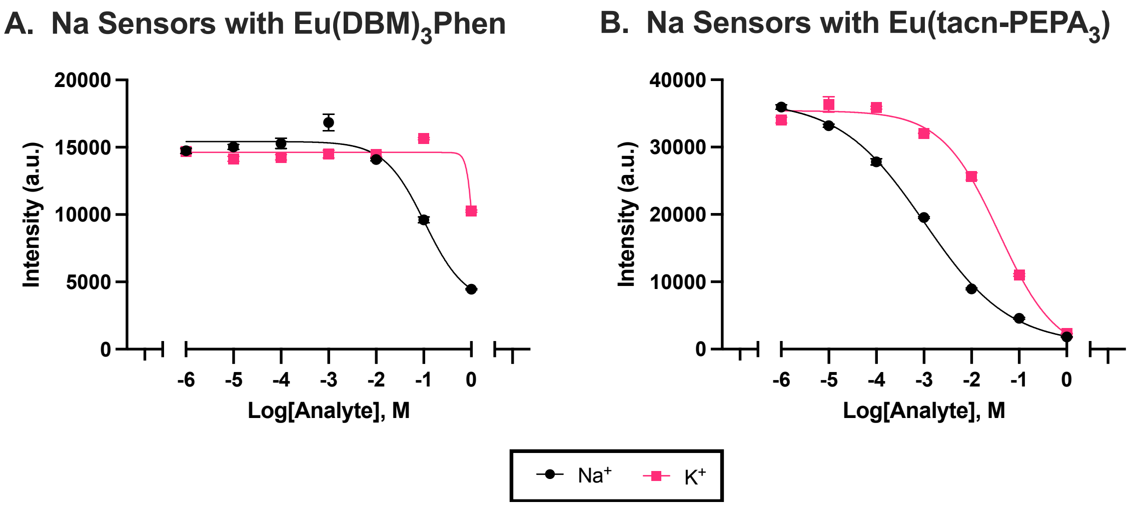

Figure S 17 Response of sodium sensors made with BB-dye and Eu-chelates (n = 3). (A) The sodium sensor incorporating Eu(DBM)$_3$Phen shows a decrease in emission intensity with increasing sodium concentration. The response is selective for sodium over potassium. (B) The sensor containing Eu(tacn-PEPA$_3$) displays a similar decreasing trend but exhibits significantly higher sensitivity to sodium. Both sensors were excited at 352 nm. Emission was monitored at 614 nm for Eu(DBM)$_3$Phen and 620 nm for Eu(tacn-PEPA$_3$). Where not visible, error bars are smaller than the data points.

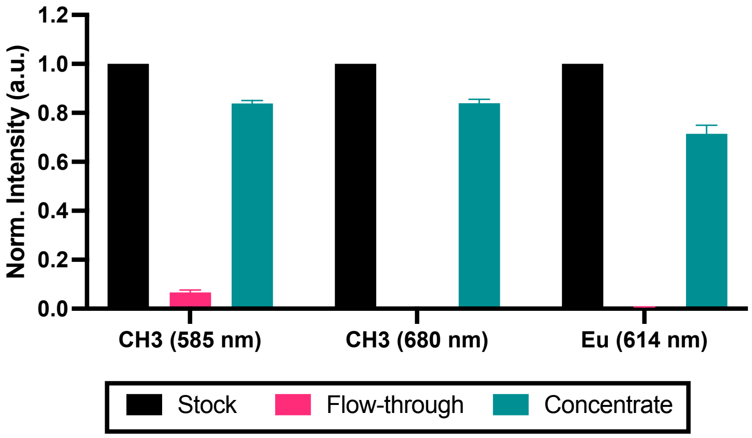

Figure S 18: Dye retention of potassium sensors fabricated with Eu(DBM)$_3$Phen (n = 3). All responses were normalized to the stock sensor solution. Both CH3 and the Eu-chelate show good retention within the sensor, as the signals from the concentrate/retentate are comparable to those of the stock. A small signal is observed in the flow-through for CH3 at 585 nm, which is likely due to background signal from the H/T buffer used in the flow-through. CH3 was excited at 500 nm and 650 nm, with emissions monitored at 585 nm and 680 nm, respectively. The Eu-chelate was excited at 352 nm, and its emission was recorded at 614 nm. Where not visible, error bars are smaller than the data points.

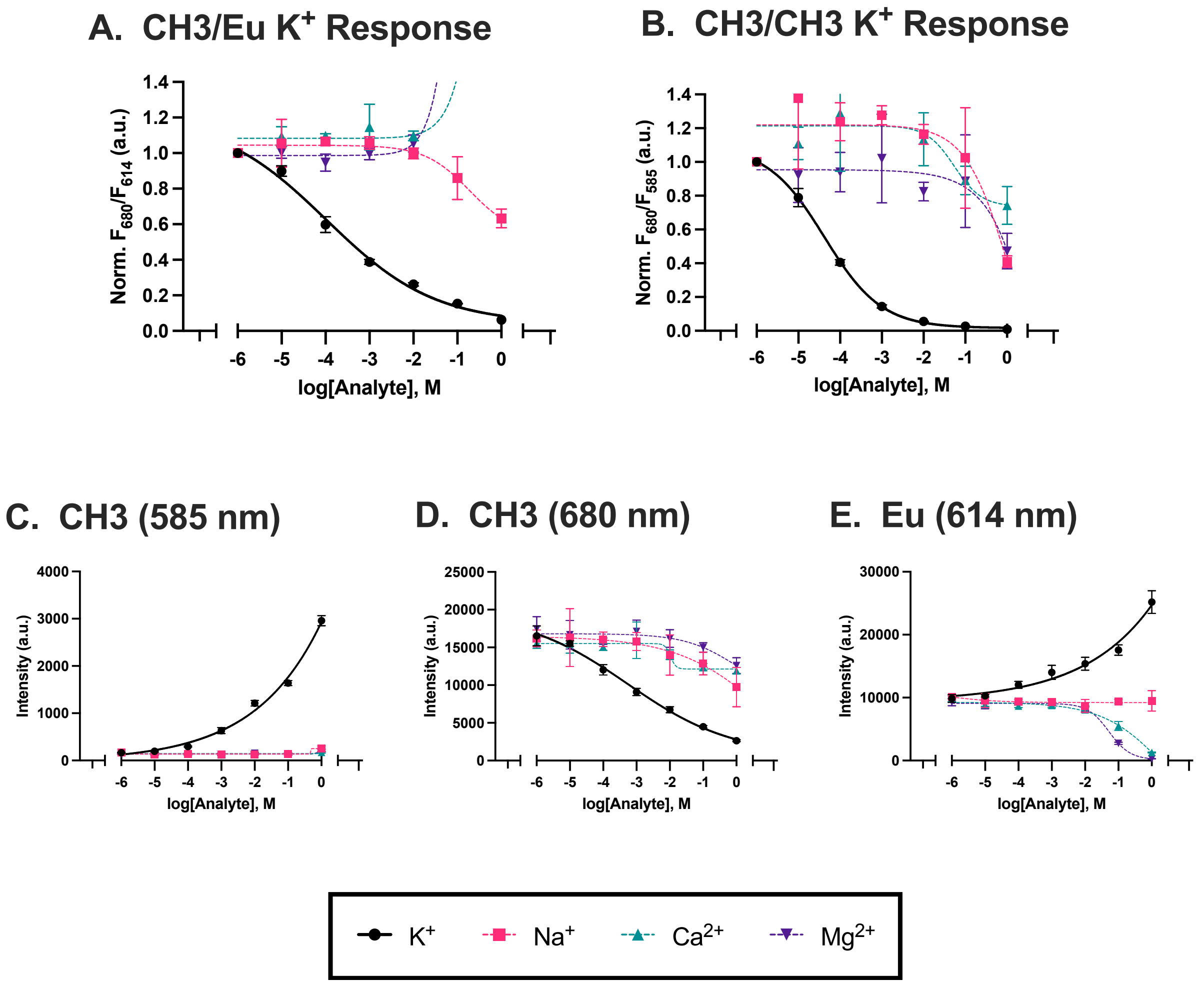

Figure S 19: Response of potassium nanosensors containing Eu(DBM)$_3$Bipy to various cations (n = 3). (A) The normalized ratiometric signal of CH3/Eu (680 nm / 614 nm) shows strong selectivity for potassium over other competing ions. This trend is further supported by the data in panels (C), (D), and (E). (B) The normalized ratiometric signal of CH3/CH3 (680 nm / 585 nm) also demonstrates potassium selectivity, consistent with the signal shifts observed in panels (D) and (E). (C) Emission profile of CH3 with excitation at 500 nm. Where not visible, error bars are smaller than the data points. (D) Emission profile of CH3 with excitation at 650 nm. (E) Emission profile of the Eu-chelate (λ_{ex} = 352 nm), showing a selective response to potassium.

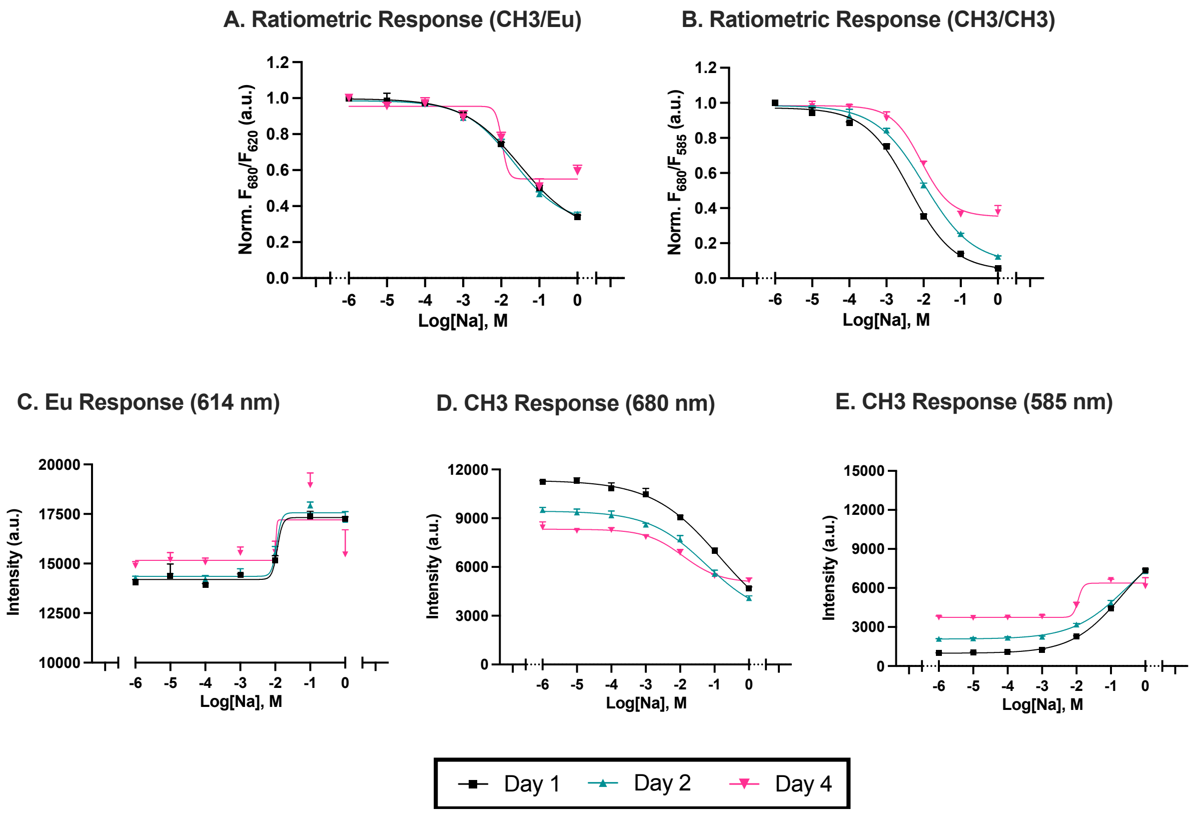

Figure S 20: Functional stability of sodium nanosensors containing Eu(tacn-PEPA$_3$) over 4 days (n = 3). (A) The normalized ratiometric signal of CH3/Eu (680 nm / 614 nm) shows good stability over the 4-day period. However, the sigmoidal fit is affected by the day 4 data point at 1 M Na^+, as seen in panels (C), (D), and (E). (B) The normalized ratiometric signal of CH3/CH3 (680 nm / 585 nm) shows a gradual drift over time, likely due to the CH3 signals at 680 nm and 585 nm changing at different rates. This trend is supported by the emission profiles in panels (D) and (E). (C) Emission profile of the Eu-chelate (λ_{ex} = 352 nm), showing no significant change over the first two days, but a higher signal at day 4. (D) Emission profile of CH3 excited at 650 nm, showing a decrease in intensity over 4 days. (E) Emission profile of CH3 excited at 500 nm, showing a gradual increase in signal over 4 days, opposite to the trend in panel (D). Where not visible, error bars are smaller than the data points.

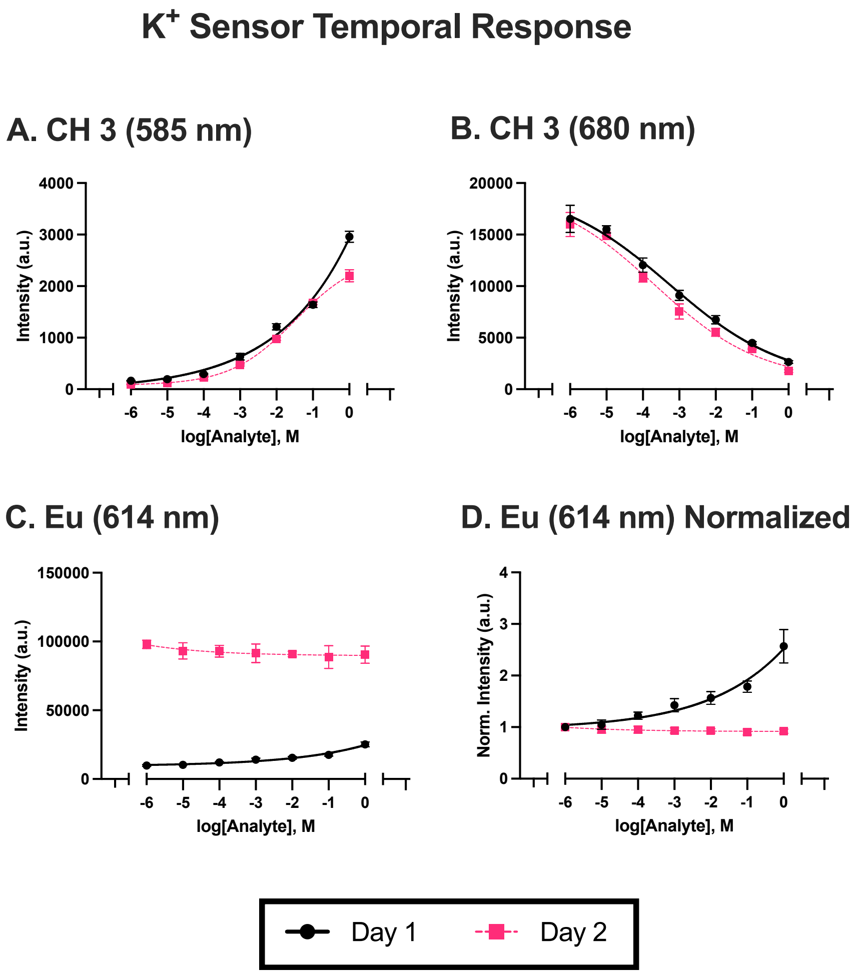

Figure S 21: Temporal response of potassium sensors containing Eu(DBM)$_3$Bipy after 2 days (n = 3). (A) and (B) show the emission response of CH3 at 585 nm (λ_{ex} = 500 nm) and 680 nm (λ_{ex} = 650 nm), respectively, with no significant change observed after 2 days. (C) and (D) display the response of Eu(DBM)$_3$Bipy (λ_{ex} = 352 nm, λ_{em} = 614 nm). Panel (C) shows the non-normalized signal, which increases after 2 days. However, when normalized to the response at 1 µM K^+ in panel (D), no significant change is observed, indicating that the sensor performance is unaffected by dye leaching or degradation. Where not visible, error bars are smaller than the data points.

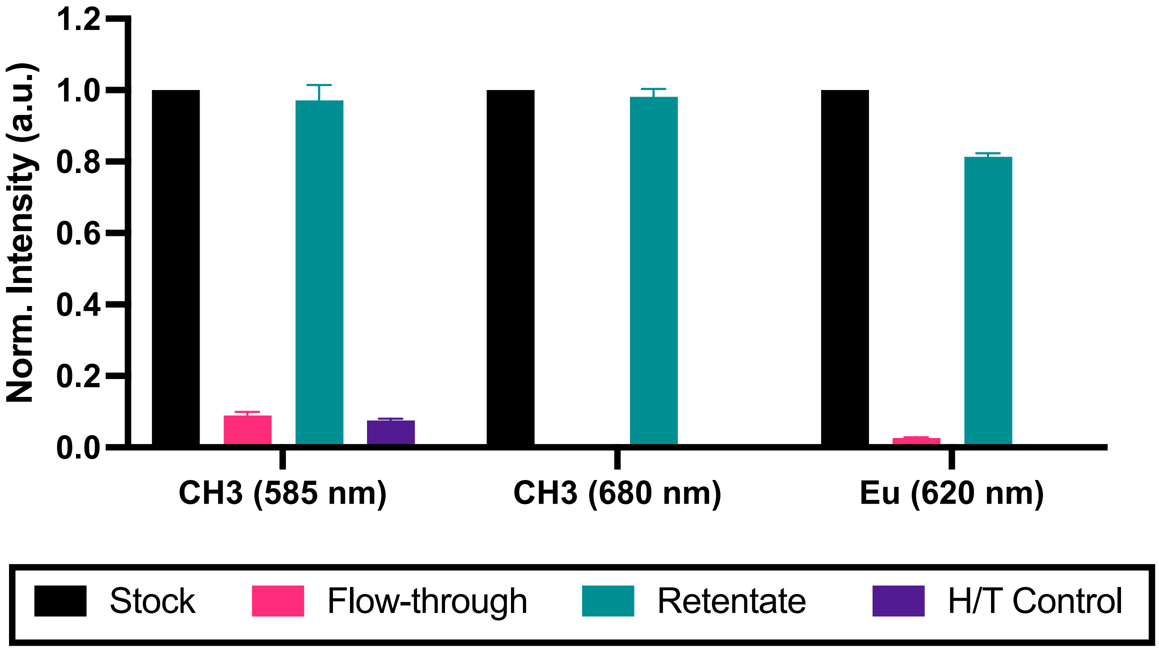

Figure S 22: Dye retention of the sodium sensors fabricated with Eu(tacn-$PEPA_3$) (n=3). All the responses were normalized to that of the stock sensor solution. Both CH3 and the Eu-chelate show good retention within the sensor, as the signals of the concentrate/retentate and the stock are similar. There is a small signal in the flow through for the CH3 when profiled at 585 nm, but this can be attributed to the signal obtained by the H/T buffer solution in the flow-through. The CH3 was excited at 500 nm and 650 nm and the emissions were observed at 585 nm and 680 nm respectively. The Eu-chelate was excited at 352 nm and its emission profiled at 614 nm. Where not visible, the error bars are smaller than that of the data points.

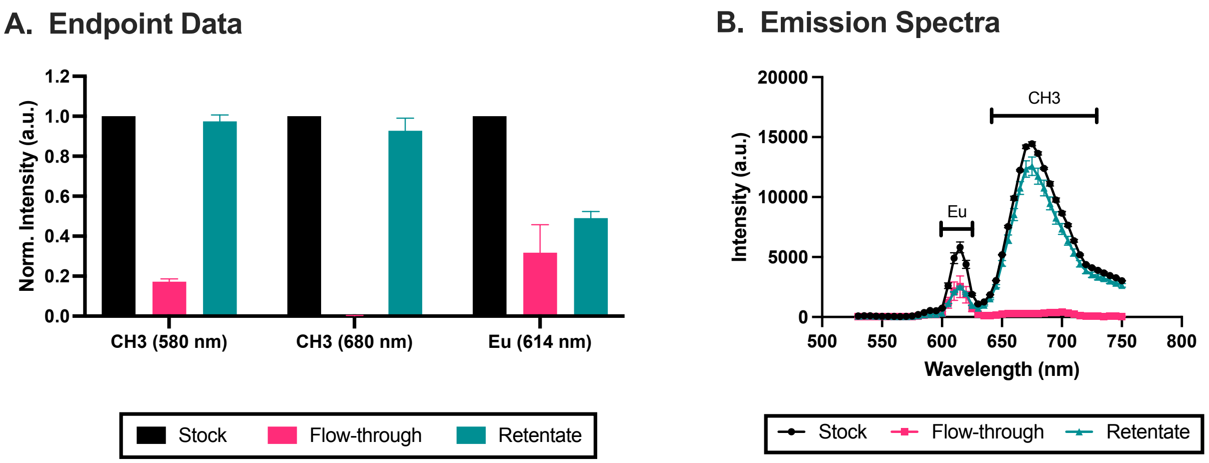

Figure S 23: Dye retention of potassium sensors fabricated with Eu(DBM)$_3$Bipy (n = 3). All responses were normalized to the stock sensor solution. (A) Endpoint data show that CH3 exhibits good retention within the sensor, as the signals from the concentrate (retentate) and the stock are similar. A small signal is observed in the flow-through at 585 nm for CH3, which is likely due to background signal from the H/T buffer in the flow-through. In contrast, the Eu-chelate shows poor retention. The retentate signal is low, and the flow-through contains a noticeable increase in signal, suggesting dye leaching or poor encapsulation. (B) This observation is further supported by the emission spectra. The CH3 signal (λ_{ex} = 500 nm and 650 nm; λ_{em} = 585 nm and 680 nm) shows strong overlap between the stock and retentate. However, the Eu-chelate signal (λ_{ex} = 352 nm; λ_{em} = 614 nm) is largely absent in the retentate and clearly present in the flow-through, confirming significant loss of the Eu complex. Where not visible, error bars are smaller than the data points
